# Special issue Heidelberg Heart II: Abstracts of oral and poster presentations

**DOI:** 10.1007/s00441-012-1412-x

**Published:** 2012-04-25

**Authors:** Werner W. Franke

**Affiliations:** German Cancer Research Center, Im Neuenheimer Feld 280, Heidelberg, 69120 Germany

**Special Helmholtz Workshop — Heidelberg Heart II**

**German Cancer Research Center, Heidelberg, Germany**

**9–11 September 2011**

**Cell and Molecular Biology of the Junctions and their Functions in Heart Tissues — When Cardiology meets Molecular Biology**

Organizers: Walter Birchmeier, Werner W. Franke
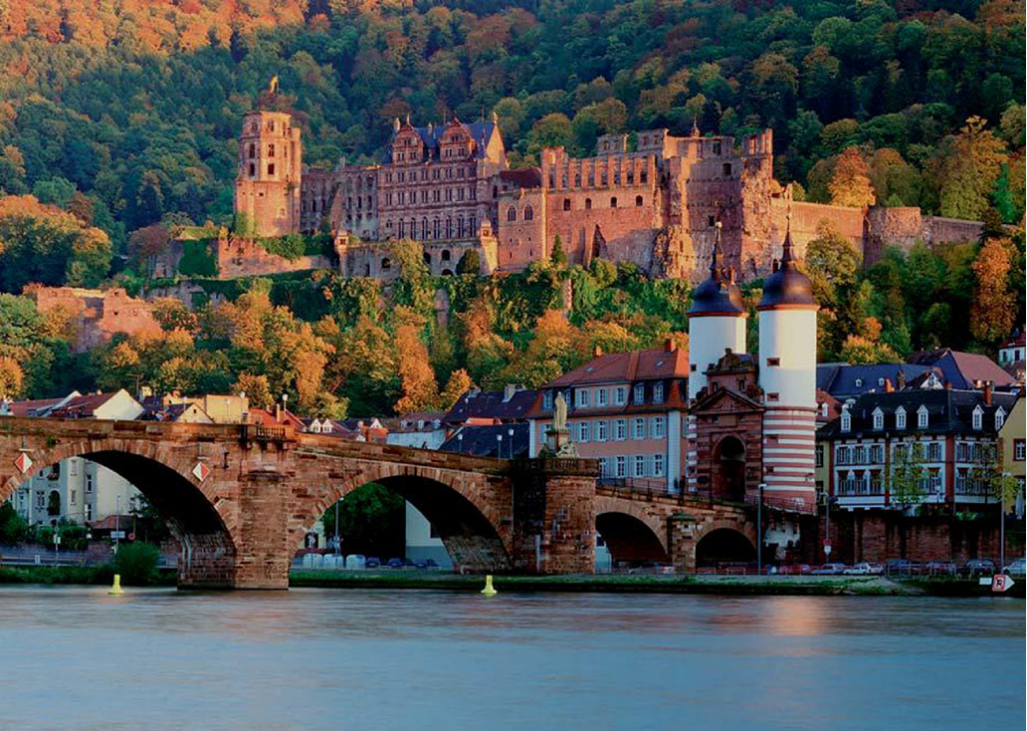



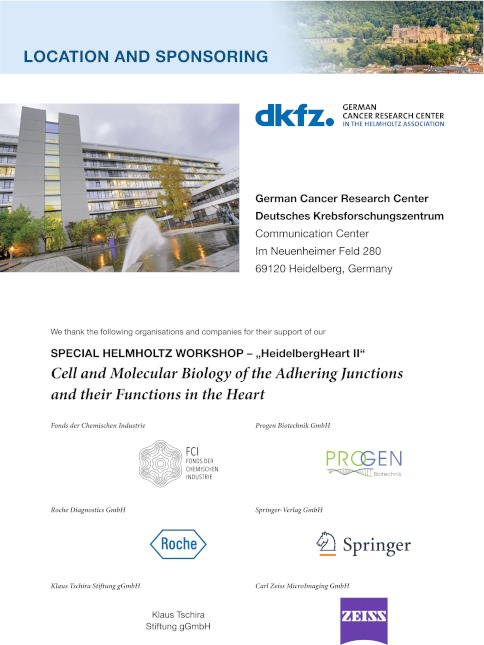


**Speakers**

**Cristina Basso** (Padua, Italy) - A 15

**Joyce Bischoff** (Boston, USA) - A 38

**Orest W. Blaschuk** (Montreal, Canada) - A 27

**Patrice Bouvagnet** (Lyon, France) - A 20

**Jonathan T. Butcher** (Ithaca, USA) - A 37

**Hugh M. Calkins** (Baltimore, USA) - A 24

**Yassemi Capetanaki** (Athens, Greece) - A 8

**Adrian H. Chester** (Harefield, UK) - A 36

**Elisabeth Ehler** (London, UK) - A 5

**Bernd K. Fleischmann** (Bonn, Germany) - A 9

**Norbert Frey** (Kiel, Germany) - A 4

**Michael H. Gollob** (Ottawa, Canada) - A 19

**Robert G. Gourdie** (Charleston, USA) - A 6

**Kathleen J. Green** (Chicago, USA) - A 1

**Axel Haverich** (Hannover, Germany) - A 35

**David P. Kelsell** (London, UK) - A 33

**Paulus Kirchhof** (Münster, Germany) - A 21

**Peter Kohl** (Harefield, UK) - A 29

**Calum A. MacRae** (Boston, USA) - A 23

**Roger R. Markwald** (Charleston, USA) - A 31

**Takashi Mikawa** (San Francisco, USA) - A 10

**Antoon F. M. Moorman** (Amsterdam, The Netherlands) - A 11

**John J. Mullins** (Edinburgh, UK) - A 25

**Sebastian Pieperhoff** (Edinburgh, UK) - A 12

**Laurentiu M. Popescu** (Bucharest, Romania) - A 30

**Karen E. Porter** (Leeds, UK) - A 28

**Nikos Protonotarios** (Naxos, Greece) - A 18

**Glenn L. Radice** (Philadelphia, USA) - A 3

**Steffen Rickelt** (Heidelberg, Germany) - A 26

**Mark W. Russell** (Ann Arbor, USA) - A 7

**Jeffrey E. Saffitz** (Boston, USA) - A 16

**Maya Simionescu** (Bucharest, Romania) - A 13

**Gaetano Thiene** (Padua, Italy) - A 22

**Adalena Tsatsopoulou** (Naxos, Greece) - A 18

**Jolanda van Hengel** (Ghent, Belgium) - A 2

**J. Peter van Tintelen** (Groningen, The Netherlands) - A 14

**Sir Magdi H. Yacoub** (Harefield, UK) - A 34

**Katherine E. Yutzey** (Cincinnati, USA) - A 32

**Abstracts of oral presentations**

**A 1 — Desmosomal molecules in and out of junctions**

Kathleen J. Green, Adi Dubash, Lisa M. Godsel

Departments of Pathology and Dermatology, Northwestern University Feinberg School of Medicine, Chicago, IL, USA

kgreen@northwestern.edu

Desmosomes are intercellular junctions that anchor the intermediate filament (IF) cytoskeleton to sites of strong intercellular adhesion, and play a critical role in ensuring mechanical integrity of the skin and the heart. Desmosome building blocks come primarily from three protein families. Transmembrane members of the cadherin family, the desmogleins and desmocollins, cooperate to form the adhesive interface. Within the junctional plaque, the cytoplasmic tails of the cadherins provide a scaffold for armadillo family members including plakoglobin and plakophilins (PKPs) 1-3 and the IF-binding protein, desmoplakin (DP), which in turn anchors the strain-bearing IF cytoskeleton to the plaque. In cardiac muscle, desmosome molecules are major architectural components of the intercalated discs, highly organized regions of the plasma membrane comprising components of adherens junctions, gap junctions, and desmosomes that together coordinate mechanical and electrochemical signaling between adjacent cardiac myocytes. In vertebrates, postnatal remodeling of these specialized regions of the plasma membrane occurs, giving rise to the area composita in which desmosomal building blocks are intermixed with components of adherens junctions.

The crucial functions of desmosome molecules in epithelial and cardiac tissues are highlighted by the discovery of mutations that cause skin and heart disease. While interference with the architectural roles of desmosome molecules has been assumed to make an important contribution to tissue responses that lead to disease pathogenesis, functions that transcend their well-established roles in adhesion and IF-anchorage are emerging. Desmosome molecules have recently been shown to guide the remodeling of microtubules during epidermal morphogenesis, and also govern actin remodeling by regulating Rho GTPases during junction assembly.

Of particular interest is the desmosomal armadillo protein plakophilin 2 (PKP2), which is reported as the most frequent target for mutation in arrhythmogenic right ventricular cardiomyopathy (ARVC), a leading cause of sudden cardiac death in the young. We previously showed that in epithelial cells, PKP2 regulates the localization and activity of RhoA to locally control actomyosin contractile signaling important for cell junction formation, while keeping global RhoA signaling in check. In the heart, signaling through RhoA and its downstream effectors is critical for normal cardiac development and physiology. Indeed, our data show that PKP2 silencing results in elevated RhoA and disruption of actin organization in cardiac myocytes. Further, loss of PKP2 or its fellow armadillo protein plakoglobin leads to elevated expression of target genes of the transcriptional regulator SRF (serum response factor), which is activated in a RhoA-dependent fashion, and along with Rho plays essential roles in cardiac development, hypertrophy and fibrosis. These data suggest a new cellular pathway through which desmosome deficiency could contribute to pathogenesis in ARVC.

**A 2 — Alpha-catenins: emerging targets for diseases**

Jolanda van Hengel ^1^, Jifen Li^2^, Steven Goossens^1^, Erhe Gao^2^, Lan Cheng^2^, Koen Tyberghein^1^, Xiying Shang^2^, Riet De Rycke^1^, Frans van Roy^1^, and Glenn L. Radice^2^

(1) Department for Molecular Biomedical Research, Flanders Interuniversity Institute for Biotechnology (VIB) and Ghent University, B-9052, Ghent, Belgium

(2) Center for Translational Medicine, Department of Medicine, Thomas Jefferson University, Philadelphia, PA, USA

Jolanda.vanhengel@dmbr.vib-ugent.be

The adherens junction (AJ) is a type of cell–cell junction that develops near the apical surface of polarized epithelial cells. Together with a bundle of cortical actin filaments, the AJ is organized as a molecular belt. The AJ comprises cadherins, catenins, and other associated proteins. Cadherins interact homophilically via their extracellular domain, and physically link the adjacent cell membranes. The cytoplasmic region of classic cadherins binds β-catenin, which in turn associates with α-catenin. Alpha-catenin is indispensable for cadherin-mediated cell adhesion. In the absence of α-catenin, the AJ is disrupted, and the apical actin belt dissociates from the cadherin–catenin complex. It was commonly believed that the cadherin–catenin complex is physically linked to actin fibers via α-catenin, and that this linkage is crucial for maintenance of the AJ. However, this model has been challenged by the recently revealed inability of the reconstructed cadherin–catenin complex to directly bind actin filaments in vitro (Drees et al. 2005; Yamada et al. 2005). However, a number of mechanisms can be proposed to reconcile these observations. One possibility is that additional mediators serve as a bridge between the cadherin-bound α-catenin and F-actin. Three homologous α-catenin proteins are known: the ubiquitously expressed αE-catenin, the neurally expressed αN-catenin, and αT-catenin with a restricted expression pattern. AlphaT-catenin is especially abundant in heart tissue, where it is co-expressed with αE-catenin in *areae compositae*, which are present at the intercalated discs (ICD). In vitro, αN- and αT-catenin can substitute for the adhesive functions of αE-catenin, but their restricted expression patterns in vivo indicate that they have tissue-specific functions. This hypothesis was confirmed by the inability of endogenous αT-catenin expression to rescue a heart-specific αE-catenin knock-out in mice (Sheikh et al. 2006). We have shown that αT-catenin has the following isoform-specific function: it interacts specifically with plakophilins (PKP), whereas αE- and αN-catenin cannot (Goossens et al. 2007). To investigate the function of alphaT-catenin, we generated a loss-of-function model in mouse. AlphaT-catenin-null mice are viable, but they exhibit progressive cardiomyopathy (Li et al. 2011). We observed reduced expression of PKP-2 in the ICD of αT-catenin-null myocardium. Furthermore, Cx43 was reduced at the ICD, including its co-localization with N-cadherin. This finding suggests that αT-catenin may function synergistically with desmosomal PKP-2 to stabilize gap junctions at the *area composita*. This gap junction remodeling was associated with an increased incidence of ventricular arrhythmias in αT-catenin-null mice when subjected to acute ischemia.

Drees F, Pokutta S, Yamada S, Nelson WJ, Weis WI (2005) α-Catenin is a molecular switch that binds E-cadherin-β-catenin and regulates actin-filament assembly. Cell 123:903–915

Goossens S, Janssens B, Bonné S, De Rycke R, Braet F., van Hengel J, van Roy F (2007) A unique and specific interaction between αT-catenin and plakophilin-2 in the area composita, the mixed-type junctional structure of cardiac intercalated discs. J Cell Sci 120:2126–2136

Li J, Goossens S, van Hengel J, Gao E, Cheng L, Tyberghein K, Shang X, De Rycke R, van Roy F, Radice GL (2012) Loss of alphaT-catenin alters the hybrid adhering junctions in the heart and leads to dilated cardiomyopathy and ventricular arrhythmia following acute ischemia. J Cell Sci 125:1058–1067

Sheikh F, Chen Y, Liang X, Hirschy A, Stenbit AE, Gu Y, Dalton ND, Yajima T, Lu Y, Knowlton KU, Peterson KL, Perriard J-C, Chen J (2006) α-E-catenin inactivation disrupts the cardiomycyte adherens junction, resulting in cardiomyopathy and susceptibility to wall rupture. Circulation 114:1046–1055

Yamada S, Pokutta S, Drees, F, Weis WI, Nelson WJ (2005) Deconstructing the cadherin-catenin-actin complex. Cell 123:889–901

**A 3 — Catenins: multi-function proteins involved in cell adhesion, communication, and signaling in the heart**

Jifen Li^1^, David Swope^1^, Lan Cheng^1^, Erhe Gao^1^, Natalia Raess^2^, Eliane J. Müller^2^, Jolanda van Hengel^3^, Frans van Roy^3^, Glenn L. Radice ^1^

(1) Center for Translational Medicine, Department of Medicine, Thomas Jefferson University, Philadelphia, PA, USA

(2) Molecular Dermatology, Institute of Animal Pathology, University of Bern, Bern, Switzerland

(3) Department of Molecular Biomedical Research, Flanders Interuniversity Institute for Biotechnology (VIB)-Ghent University, B-9052, Ghent, Belgium

Glenn.Radice@jefferson.edu

Efficient cardiac contractile function is highly dependent upon the coordinated mechanical and electrical activation of the myocardial tissue. Many of the molecular components required for mechano-electrical coupling in the heart are localized at the end-to-end connection between myocytes called the intercalated disc (ID). The ID consists of three main junctional complexes: adherens junctions and desmosomes provide strong cell–cell adhesion, and gap junctions provide electrical coupling between the myocytes. ID structure is dependent on N-cadherin, the only classical cadherin expressed in the myocardium (Kostetskii et al. 2005). Cadherin-mediated adhesion requires interaction with the actin cytoskeleton via a family of proteins called catenins. Whether the different catenin subtypes have distinct or synergistic functions in the heart is not clear.

Mutations in γ-catenin (also known as plakoglobin) are associated with arrhythmic right ventricular cardiomyopathy (ARVC), a hereditary heart-muscle disease that causes sudden cardiac death (SCD) in young people. To investigate the role of PG in ARVC, we generated a cardiac-specific knockout (CKO) of the *plakoglobin* (*JUP*) gene in mice (Li et al. 2011). Interestingly, despite gap junction remodeling, PG CKO mice have no apparent conduction abnormality and survive longer than expected. Importantly, the PG homolog, β-catenin, showed increased association with the gap junction protein, connexin43 (Cx43) in PG CKO hearts. To determine whether β-catenin protects PG CKO animals from sudden arrhythmic death, we generated mice lacking both PG and β-catenin specifically in the heart (i.e., double knockout, DKO). The PG/β-catenin DKO mice exhibited acute cardiomyopathy, fibrous tissue replacement, and conduction abnormalities resulting in SCD 3–5 months after deleting both genes. In contrast to the PG and β-catenin single mutants, N-cadherin was significantly reduced at the ID in the PG/β-catenin DKO mice. Moreover, the ID structure was disrupted in the PG/β-catenin DKO hearts, consistent with the loss of adherens junction and desmosome proteins from the ID. Collectively, our results demonstrate that PG is more important for the structural integrity of the heart than β-catenin; however, β-catenin maintains gap junctions in the absence of PG, indicating that these proteins cooperate to maintain electrical coupling in the heart.

There are two α-catenin subtypes expressed in the myocardium, αE-catenin and αT-catenin; together, they are thought to modulate interactions between N-cadherin/catenin complex and the actin cytoskeleton. A novel αT-catenin KO mouse model demonstrates for the first time how perturbation in αT-catenin can affect both PKP-2 and Cx43, thus highlighting the importance of understanding the cross-talk between the junctional proteins of the area composita and its implications for arrhythmogenesis (Li et al. 2012).

Kostetskii I, Li J, Xiong Y, Zhou R, Ferrari VA, Patel VV, Molkentin JD, Radice GL (2005) Induced deletion of the N-cadherin gene in the heart leads to dissolution of the intercalated disc structure. Circ Res 96:346–354

Li J, Swope D, Raess N, Cheng L, Muller EJ, Radice GL (2011) Cardiac tissue-restricted deletion of plakoglobin results in progressive cardiomyopathy and activation of β-catenin signaling. Mol Cell Biol 31:1134–1144

Li J, Goossens S, van Hengel J, Gao E, Cheng L, Tyberghein K, Shang X, De Rycke R, van Roy F, Radice GL (2012) Loss of alphaT-catenin alters the hybrid adhering junctions in the heart and leads to dilated cardiomyopathy and ventricular arrhythmia following acute ischemia. J Cell Sci 125:1058–1067

**A 4 — Transgenic overexpression of the intercalated disc protein myozap causes protein aggregate-associated cardiomyopathy**

Derk Frank^1^, Thalia S. Seeger^2^, Claudia Rohr^2^, Christian Kuhn^1^, Christine Grund^3^, Rainer Will^2^, Werner W. Franke^3^, Hugo A. Katus^2^, Norbert Frey ^1^

(1) Dept. of Cardiology and Angiology, UK-SH, Kiel, Germany

(2) Dept. of Internal Medicine III, University of Heidelberg, Germany

(3) Helmholtz Group Cell Biology, DKFZ, Heidelberg, Germany

Norbert.Frey@uk-sh.de

The intercalated disc (ID) is an important component of the cell–cell contact structures of cardiomyocytes. During the last decade, it became evident that the molecular components of the ID are critical regulators in the pathogenesis of inherited cardiac disease (van Tintelen et al. 2007). We were able recently to identify and characterize a novel cardiac-enriched ID protein, termed myozap. It interacts with several other ID proteins including desmoplakin.

Mechanistically, it represents a positive modulator of the Rho-dependent SRF pathway linking the ID to gene regulation processes and actin dynamics (Seeger et al. 2010). To further insight in myozap’s function in vivo, we generated a mouse model with cardiac-restricted overexpression of myozap cDNA using the αMHC promoter. These mice developed substantial cardiac hypertrophy (heart weight/tibia length +43 %, *p* < 0.01, *n* = 8–10) as well as progressive LV dilation (LVEDD +20 %, *p* <  0.01). Consistently, myozap-transgenic hearts displayed upregulation of the hypertrophy-associated “fetal” gene program (e.g., ANF 4.3-fold, *p* <  0.001, and BNP 2.2-fold, *p* <  0.01, *n* = 7–9). Next, myozap transgenic animals were subjected to various forms of stress, including voluntary running wheel exercise, which led to an accelerated cardiomyopathy with premature LV dysfunction and dilation. Moreover, chronic infusion of the alpha-agonist phenylephrine (PE) caused significant lethality in myozap-Tg [*n* = 5/8, *p* <  0.05 vs WT (1/7)]. Unexpectedly, on the ultrastructural level, we were able to detect bulky protein aggregates containing myozap, desmoplakin, and other ID proteins. This aggregate-associated pathology closely resembled the changes in the hearts of patients suffering from desminopathies (Goldfarb and Dalakas 2009). It should be noted that desmin was not detectable in the aggregates of myozap tansgenic mice, but was displaced from the ID.

Taken together, cardiac overexpression of the novel ID protein myozap leads to hypertrophy and dilated cardiomyopathy, accompanied by protein aggregates and associated with sudden death upon PE treatment. Further analyses of this transgenic model may help to understand the pathogenesis and pathophysiology of protein aggregate-associated cardiomyopathies.

Goldfarb LG, Dalakas MC. (2009) Tragedy in a heartbeat: malfunctioning desmin causes skeletal and cardiac muscle disease. J Clin Invest 119:1806–1813

Seeger TS, Frank D, Rohr C, Will R, Just S, Grund C, Lyon R, Luedde M, Koegl M, Sheikh F, Rottbauer W, Franke WW, Katus HA, Olson EN, Frey N (2010) Myozap, a novel intercalated disc protein, activates serum response factor-dependent signaling and is required to maintain cardiac function in vivo. Circ Res 106:880–890

van Tintelen JP, Hofstra RM, Wiesfeld AC, van den Berg MP, Hauer RN, Jongbloed JD. (2007) Molecular genetics of arrhythmogenic right ventricular cardiomyopathy: emerging horizon? Curr Opin Cardiol 22:185–192

**A 5 — The intercalated disc — a truly special type of cell-cell contact in dilated cardiomyopathy**

Alain Hirschy, Elisabeth Ehler

King’s College London BHF Research Excellence Centre, The Randall Division of Cell and Molecular Biophysics and The Cardiovascular Division, New Hunt’s House, Guy’s Campus, London SE1 1UL, United Kingdom

elisabeth.ehler@kcl.ac.uk

Two multiprotein complexes are essential for proper functioning of the heart: the myofibrils are responsible for carrying out the contractile work, and the intercalated disc, a specialised type of cell–cell contact, is indispensable for proper mechanical and electrochemical contact between the individual cardiomyocytes. Both structures are characterized by their extremely regular arrangement, which in case of the intercalated disc is only achieved after birth. We have shown previously that alterations in intercalated disc composition are a hallmark of dilated cardiomyopathy (DCM) in mice and men (Ehler et al., 2001). There is an upregulation of expression of proteins that are involved in the anchoring of actin filaments accompanied by an increased convolution of the plasma membrane. This probably leads to an overall stiffening of the cell–cell contacts. In the rodent DCM heart, there is also a reduction in the expression of gap junction proteins, which is expected to lead to decreased intercellular communication. Since stoichiometry is clearly important for proper function, we have decided to challenge it by gain and loss of function experiments, targeting the adherens junction protein beta-catenin.

In our system, the postnatal loss of beta-catenin in cardiomyocytes has comparatively little effect on heart function; however, when we overexpress beta-catenin in the heart we observe a dramatic phenotype of DCM, with all the mice dead by 5 months of age (Hirschy et al., 2010). Interestingly, despite an excess of beta-catenin per targeted cell, we never observed any beta-catenin in the nucleus of postnatal cardiomyocytes. We conclude that increased amounts of beta-catenin at the intercalated disc are not tolerated by the heart, but that nuclear beta-catenin signaling is probably not very relevant in the fully differentiated cardiomyocyte.

Ehler E, Horowits R, Zuppinger C, Price RL, Perriard E, Leu M, Caroni P, Sussman M, Perriard J-C (2001) Alterations at the intercalated disk associated with the absence of muscle LIM protein. J. Cell Biol 153:763–772

Hirschy A, Croquelois A, Perriard E, Schoenauer R, Agarkova I, Hoerstrup SP, Taketo MM, Pedrazzini T, Perriard J-C, Ehler D (2010) Stabilised beta-catenin in postnatal ventricular myocardium leads to dilated cardiomyopathy and premature death. Basic Res Cardiol 105:597–608

**A 6 — The connexin carboxyl terminus and cardiac gap junction organization**

Robert G. Gourdie

Department of Regenerative Medicine and Cell Biology and Clemson-MUSC Bioengineering Program, Medical University of South Carolina, Charleston, SC, USA

gourdier@musc.edu

The precise spatial order of gap junctions at intercalated disks in adult ventricular myocardium is thought vital for maintaining cardiac synchrony. Breakdown or remodeling of this order is a hallmark of arrhythmic disease of the heart. The principal component of gap junction (GJ) channels between ventricular cardiomyocytes is connexin43 (Cx43). Protein–protein interactions and modifications of the carboxyl-terminus (CT) of Cx43 are key determinants of GJ function, size, distribution, and organization during normal development and in disease processes. This talk will focus on the work of my lab on protein interactions at the Cx43 CT in the regulation of GJ organization. There will be a particular emphasis on the role of the protein Zonula Occludens-1 (ZO-1). Topics covered will include our recent identification of the perinexus — a novel, hemichannel-containing domain surrounding the GJ that is enriched for Cx43-ZO-1 interaction.

Our recent studies of the mode-of-action of a peptide based on the Cx43 CT that inhibits GJ remodeling at injury border zone (IBZ) and reduces inducible arrhythmias following cryo-infarction of the left ventricle will be presented. Also discussed will be preliminary data on potential assignments of the Cx43 CT in myocyte–fibroblast interaction at the IBZ and ZO-1 interaction with Cx40 and Cx45 — connexins expressed in the conduction system.

**A 7 — Outside-in and inside-out signaling through cell–cell and cell–matrix adhesion complexes promotes myofibril organization in striated muscle**

Mark W. Russell

Division of Pediatric Cardiology, Department of Pediatrics and Communicable Dieases, University of Michigan, Ann Arbor, MI, USA

mruss@med.umich.edu

During development, cardiac and skeletal myoblasts differentiate into myocytes and skeletal myotubes with mature contractile structures that are precisely oriented with respect to surrounding cells and tissues. Establishment of this highly ordered structure requires coordinate and reciprocal interactions between the differentiating myocytes and the extracellular environment, including neighboring cells and the surrounding extracellular matrix. “Outside-in” signals from the extracellular environment result in changes to myocyte morphology, and promote new myofibril assembly; in return, “inside-out” signals pattern adjoining cells and the extracellular matrix, and coordinate the assembly and organization of myofibrils across cells and tissues. Communication between the cell and extracellular environment is mediated by transmembrane adhesion complexes, the composition and complexity of which depend on the cell type and degree of differentiation. In cardiac myocytes, axial connections occur at intercalated disks in which desmosomes (maculae adhaerentes), fasciae adhaerentes, and gap junctions form mechanical and electrochemical communications between adjacent cells. Examination of remodeling adult rat cardiac myocytes in primary culture suggests that outside-in signals from surrounding cells establish a well-defined axis of contraction, resulting in cytoskeletal remodeling and reorganization of the myofibrils. Inside-out signals restrict the adhesive contacts to the termini of the reorganized myofibrils, supporting the layered addition of armadillo family proteins and cytoskeletal adaptors to the cadherin- and desmosomal cadherin--based adhesive contact. A similar process occurs at integrin-based adhesion contacts within the costameres of cardiac and skeletal muscle. At those sites, outside-in and inside-out signals transmitted through the adhesion complexes promoted organization of the internal structure of the myocyte and translation of those structural cues to surrounding cells and tissues. Our findings support a model of myofibril assembly and organization as a dynamic process involving the interaction of intracellular compartments with specialized transmembrane adhesion domains. Identification of the linking elements that promote the coordinated remodeling of the sarcomere and cell–cell and cell–matrix contacts will be essential in developing strategies to prevent muscle injury and promote muscle repair in patients with cardiac and skeletal myopathies and muscular dystrophies.

**A 8 — Desmin as a major player in heart failure and a potential link between ARVD/C and DCM**

Yassemi Capetanaki

Division of Cell Biology, Center of Basic Research I, Biomedical Research Foundation, Academy of Athens, Athens, Greece

ycapetanaki@bioacademy.gr

It has been demonstrated that gene inactivation in mice or mutations in humans in the major intermediate filament protein desmin leads to all forms of cardiomyopathy (DCM, HCM, RCM, ARVD/C) and eventually heart failure. Desmin forms a continuous network connecting the contractile apparatus to the sarcolemma (costameres & intercalated discs), nucleus, and several membranous organelles. Mutations in proteins that associate to the desmin network directly or indirectly also lead to cardiomyopathy. Importantly, the type of developed cardiomyopathy depends on the cardiomyocyte structure at which the desmin network-associated proteins are localized. Thus, costameric proteins such as dystrophin, α-dystroglycan, and δ-sarcoglycan, nuclear proteins such as the nuclear intermediate filament protein lamin A/C, and different lamin-associated proteins, such as LAP2alpha and emerin, and finally, at the Z-disc region, proteins such as plectin and the chaperon protein αB-crystallin, are associated to DCM. On the other hand, mutations in genes encoding several proteins of the *area composita* (Franke et al. 2007) of the intercalated disc, such as desmoplakin, plakoglobin, plakophilin 2, desmoglein 2, and desmocollin, have been identified as the genetic basis of ARVD/C. Interestingly, preliminary genotype–phenotype assessment indicates that mutations affecting the outer dense plaque of the desmosome result in ARVD/C, while mutations at the inner dense plaque, particularly affecting the desmin-binding site of desmoplakin, result in ARVD/C with left ventricular involvement and extensive overlap with DCM. Similarly, the S13F desmin mutation, identified recently in patients with ARVD/C (Van Spaendonck-Zwarts et al. 2011), is the most upstream mutation of the desmin head domain found so far, and the observation that this mutation results in abnormalities at the intercalated disc region, potentially by disruption of desmin-desmoplakin association, is important and could allow the molecular understanding of the transition from mainly ARVD/C to ARVDC/DCM and to mainly DCM development.

The most common desmin-related heart disease is the TNF- α linked heart failure. We have demonstrated that activation of caspases by cytokines, such as TNF-α, known to cause heart failure, leads to desmin cleavage and aggregate formation, loss of desmin and other proteins (at least β-catenin and desmoplakin) from intercalated discs (IDs), destabilization of IDs, mitochondrial defects, and heart failure. Strikingly, we have found that when the WT desmin is replaced by a caspase-resistant desmin (D263E), in TNF-α over-expressing mouse heart, these pathological features are greatly ameliorated, demonstrating that desmin is a major target and plays a crucial role in the development of the TNF-α-induced heart pathology (Panagopoulou et al. 2008). As our studies have demonstrated (reviewed by Capetanaki et al. 2007), the desmin null heart failure model possesses a combination of the pathophysiology of most types of cardiomyopathy, particularly DCM and ARVD/C (characterized by cell death, extensive inflammation, fibrosis, and degeneration, in both right and left ventricle, followed by systolic dysfunction and conduction system defects). Therefore, we use this model for several studies aimed to both unravel the mechanism of cardiomyopathy and heart failure development, as well as to develop therapeutic strategies for the disease. The successes towards these goals, and specifically the rescue of the desmin deficient heart failure by αB-cystallin overexpression, will be discussed.

Capetanaki Y, Bloch RJ, Kouloumenta A, Mavroidis M,Psarras S (2007) Muscle intermediate filaments and their links to membranes and membranous organelles. Exp Cell Res 313:2063–2076

Franke WW, Schumacher H, Borrmann CM, Grund C, Winter-Simanowski S, Schlechter T, Pieperhoff S, Hofmann I (2007) The area composita of adhering junctions connecting heart muscle cells of vertebrates - III: assembly and disintegration of intercalated disks in rat cardiomyocytes growing in culture. Eur J Cell Biol. 86:127–142

Panagopoulou P , Davos C, Milner D, Varela E, J, Mann D, Capetanaki Y (2008) Desmin mediates TNF-a induced aggregate formation and intercalated disk reorganization in heart failure. J Cell Biol 181:761–775

Van Spaendonck-Zwarts KY, van Hessem L, Jongbloed JDH, Hermien EK, de Walle HEK, Capetanaki Y, van der Kooi AJ, van Langen IM, van den Berg MP, van Tintelen JP (2011) Desmin related myopathy: a review and meta-analysis. Clin Genet 80:354–366

**A 9 — Potential and pitfalls of cellular replacement approaches in the heart**

Bernd K. Fleischmann

Institute of Physiology I, Life & Brain Center, University of Bonn, Germany

bernd.fleischmann@uni-bonn.de

Myocardial infarction is characterized by an irreversible loss of cardiomyocytes and scar formation, often resulting in heart failure. The only causal treatment currently available is heart transplantation. However, due to shortage of donor organs, cell replacement using progenitors and/or stem cells is considered a promising alternative approach. We have explored the plasticity of these cells to differentiate into cardiac muscle, their physiological integration and contribution to pump function in vivo. Heart infarctions (cryoinjury, LAD ligation) were induced in mouse and progenitors/stem cells mobilized with cytokines or directly injected into the infarct area. We have first assessed the plasticity of bone marrow (BM)-derived cells. These neither differentiated into functional cardiac muscle or endothelial cells, nor strongly enhanced left ventricular function. Similar results were obtained when using BM-derived mesenchymal stem cells. Importantly, their differentiation was not restricted by the heart tissue, as calcifications and bone formation could frequently be observed. In contrast, fetal cardiomyocytes stably engrafted into the infarct, and enhanced left ventricular function. Furthermore, the engrafted cardiac muscle cells functionally integrated into the cardiac and strongly reduced post-infarct arrhythmias. This was related to increased conduction velocities in areas containing the cellular grafts. We also analyzed the potential of embryonic stem (ES) cell-derived cardiomyocytes for cell replacement therapy. An antibiotic-based selection approach was used to obtain highly purified cardiomyocytes and to avoid teratoma formation due to contaminating pluripotent ES cells.

Transplantation experiments revealed that co-injection of purified cardiomyocytes and fibroblasts increased the degree of engraftment, and resulted in an improvement of left ventricular function. Thus, cardiac progenitors and ES cell-derived cardiomyocytes stably engraft into the infarcted myocardium and enhance heart function.

**A 10 — Cellular and molecular aspects of early heart formation**

Takashi Mikawa, Michael Bressan

Cardiovascular Research Institute, University of California, San Francisco, CA, USA

takashi.mikawa@ucsf.edu

Complex organization of the heart is established through the orchestrated and sequential processes of commitment, proliferation, and movements of multiple cell types. A spatiotemporally regulated binary decision model was once considered the major mechanism for generating multiple cardiac cells from a common stem cell population. This model was used to describe the genesis of myocyte vs endocardial endothelial cell lineages and atrial vs ventricular myocyte lineages, as well as coronary endothelial vs smooth muscle lineages. Contrary to this idea, mounting evidence collected in recent years indicates that lineage segregation of individual cardiac cell types actually occurs prior to arrival of their progenitors to the heart. The resolution of these two models lies at the core of many controversies in cardiovascular developmental biology. They need not be mutually exclusive, however. For instance, differing components of the cardiac conduction system rely on both predetermination of separate cell population and diversification of fate from common precursors. We have shown that the induction and patterning of the fast-conducting Purkinje fiber network, which is mediated by hemodynamic-activated paracrine cues, involves the recruitment of already beating ventricular myocytes into the Purkinje fiber lineage. Conversely, our recent data suggest that prior to heart tube formation the pacemaker cell lineage of the sinoatrial node is specified in a mesoderm population that is separate from precursors of other cardiac cell types. Our cell lineage studies in chick embryos have also demonstrated that the segregation as well as differentiation of coronary smooth muscle, fibroblast, and endothelial cell lineages begins in an extracardiac tissue, the proepicardium, prior to their entry to the heart. A separate origin of coronary smooth muscle and endothelial cell lineages have been confirmed by recent genetic studies of mouse embryos. Thus, a precisely timed sequence of development and integration of individual cardiac sub-components is mediated through multiple genetic and epigenetic programming during heart formation in higher vertebrates.

[Supported in part by grants from NIH-NHLBI.]

**A 11 — Development of the building plan of the heart**

Antoon F.M. Moorman

Department of Anatomy, Embryology & Physiology, Academic Medical Centre, Amsterdam, The Netherlands

a.f.moorman@amc.uva.nl

One of the most fascinating aspects in the formation of the heart is the very early development of the electrical patterning as can be registered by the ECG, which is the registration of the rhythmic waves of depolarizing activity over the cardiac muscle. In the mature heart, the conduction system is held responsible for the rhythmic excitations and contractions. However, in chicken embryos a sinusoidal type of ECG can already be derived from the linear heart tube stages at about 2 days of development onward; and less than 1 day later, when chamber formation has just been initiated, an adult type of ECG can be monitored. The presence of an adult type of ECG in these early embryonic hearts betrays the development of fast-conducting chambers rather than the presence of a conduction system. We now know that the primary heart tube as seen in the early embryo contains the precursors for the left ventricle only, or even less, whereas the precursor cells for the remainder of the cardiac components are continuously added to both the venous and arterial pole of the heart tube during further development from a single center of growth outside the heart. Therefore, it is impossible that the straight heart tube contains the precursors for the conduction system as rings separating the purported cardiac segments. While the primary heart tube is growing by addition of cells, it does not show significant cell proliferation until chamber differentiation and expansion starts locally in the tube. The transcriptional repressors Tbx2 and Tbx3 locally repress the chamber-specific program of gene expression, by which these regions are allowed to differentiate into the distinct components of the conduction system. The cardiac building plan and the underlying mechanisms of its formation are conserved from fish to man.

Detailed reconstructions of the developmental patterns of expression of Tbx3 during development in mouse and human have revealed that Tbx3 is expressed in those areas of the heart tube that do not become chamber, i.e., in the sinu-nodal region, internodal region, atrioventricular junction, atrioventricular bundle, and bundle branches. These areas comprise not only the conventional conduction system, but also the highly controversial areas of the internodal region and the entire atrioventricular junction. Also the (right) ventricular outflow tract initially expresses these transcriptional repressors, preventing it from chamber differentiation. These observations provide an embryonic basis for why some areas in the heart are more arrhythmogenic than other regions.

Christoffels VM Moorman AFM (2009) Development of the cardiac conduction system: Why are some regions of the heart more arrhythmogenic than others? Circulation 2:195–207

Moorman AFM, Christoffels VM (2003) Cardiac chamber formation: development, genes, and evolution. Physiol Rev 83:1223–1267

Sizarov A, Ya J, PhD, de Boer BA; Lamers WH, Christoffels VM, Moorman AFM (2011) Formation of the building plan of the human heart: morphogenesis, growth, and differentiation. Circulation 123:1125–1135

van den Berg G, Abu-Issa R, de Boer BA, Hutson MR, de Boer PAJ, Soufan AT, Ruijter JM, Kirby ML, van den Hoff MJB, Moorman AFM (2009) A caudal proliferating growth center contributes to both poles of the forming heart tube. Circ Res 104:179–188 (On-line interactive 3D PDF file available)

**A 12 — Modulations of cardiomyocyte adhering junctions during embryonic development and evolution**

Sebastian Pieperhoff

Centre for Cardiovascular Science, Queen's Medical Research Institute, University of Edinburgh, 47 Little France Crescent, Edinburgh, EH16 4TJ, Scotland, UK

S.Pieperhoff@gmail.com

A detailed reinvestigation of adult mammalian cardiomyocyte adhering junctions resulted in the discovery and definition of the composite junction (*area composita*). The chosen terminology highlights the hybrid character of adhering junctions connecting adult mammalian cardiomyocytes, which are composed of typical desmosomal as well as adherens junction components. Therefore, desmosomal proteins are not restricted to relatively small desmosomes (as in various epithelia), but are localized within the entire cardiac intercalated disks.

Obviously, desmosomal proteins serve their function also in adhering junctions anchoring myofibrillar actin bundles, the composite junctions. This is of particular interest, as single mutations in genes encoding for such desmosomal proteins have been linked to the development of inherited forms of cardiomyopathies, such as the arrhythmogenic right ventricular cardiomyopathy/dysplasia (ARVC/D), often without any signs of cutaneous disease involvement. In a subsequent series of studies, I was then able to show that the formation of composite junctions is a rather late event in both, mammalian development and vertebrate evolution. Nascent cardiomyocytes of early mammalian embryonic stages are for the most part connected by relatively small intermediate filament binding desmosomes and myofibrillar actin binding adherens junctions (quite similar to adhering junctions connecting epithelial cells). During late heart development (after birth), single components of both structures become more and more fused, and gradually relocated at the two poles within the composite junctions of the mature mammalian ventricular cardiomyocytes. Furthermore, results of studies on the adhering junctions connecting cardiomyocytes of various lower vertebrate and invertebrate species will be briefly presented. The findings described will be discussed in relation to cardiomyopathy development and heart muscle regeneration.

**A 13 — Endothelia of heart vessels: structure, molecules, functions and dysfunctions**

Maya Simionescu

Institute of Cellular Biology and Pathology "Nicolae Simionescu", Bucharest, Romania

maya.simionescu@icbp.ro

The endothelial cell (EC) has earned the attention and respect of biologists, who over the years discovered that far from being a gratuitous cellophane-like layer, it has a key role in body homeostasis and a large variety of pathologies. Along the cardiovascular system, the structure of the EC layer, which constitutes the boundary between the blood and the tissues, varies as a function of the vessel or organ in which they reside. The acquired innate heterogeneity consisting of the distribution/number and organization of caveolae, channels, type of intercellular junctions, and the presence/absence of fenestrae, confers to ECs the ability to adjust, sense, monitor, command, and modulate particular local functions, as well as to respond to a variety of aggressive factors. In physiological conditions, EC heart vessels (and in many other locations) have a large array of constitutive functions and the proper molecular configuration to regulate the bidirectional receptor-mediated and receptor-independent transcytosis of molecules between the plasma and tissues, to execute endocytosis, to synthesize their own basal lamina and extracellular matrix components, to guard vascular tone (by balanced synthesis of vasoactive mediators PGI2, NO, EDHF, and endothelin), to regulate cellular cholesterol and lipid homeostasis, and to administer haemostasis (via vWf and PAI-1), signal transduction, and immunity. At the molecular level, many of these functions are implemented by EC caveolae, which are structurally and functionally differentiated microdomains of the plasmalemma endowed with receptors for LDL, HDL, albumin, transferrin, EGF, AGE, and insulin, as well as death receptors (IL-1-R and P75-R).

Pathological conditions, i.e., exposure to cardiovascular risk factors (hyperlipemia/hyperglycemia), lead to EC dysfunctions, a key event in all stages of vascular diseases such as atherosclerosis. The ECs sense the variations occurring in the plasma or tissues, and initiate a focal response (in arterial-susceptible areas) manifested firstly by adaptation/modulation of their constitutive functions, such as increased transcytosis of β-lipoproteins (βLp), and enhanced synthesis of basal lamina and extracellular matrix components. The increased transcytosis and the reduced efflux from the intima, generate βLp alterations (oxidation, glycation, enzymatic modifications) and retention within the ECs' hyperplasic basal lamina as modified lipoproteins (mLp). The latter, together with modifications of plasma homeostasis, induce EC dysfunction (novel adhesion molecules, secretion of chemokines, cytokines), which triggers a robust inflammatory reaction (recruitment and transvasation of immune cells within the intima). All these alterations contribute to atheroma formation and development in the heart vessels, aorta, and other arterial segments. Ultimately, the insults present inside and outside the vessel wall lead to EC injury, apoptosis, and the ensuing vulnerable plaque rupture and atherothrombosis. At the molecular level, the disease lies within the EC, since vascular aggressors induce a cell organelle pathology manifested by amendments of membrane components (new adhesion molecules), malfunction of caveolae, and alterations in regulated transcytosis, and in the functions of the endoplasmic reticulum, Golgi complex (switch to a secretory phenotype), lysosomes (enzyme failure), and opening of intercellular junctions. The wealth of data on the role of ECs in health and disease may constitute together a complex discipline that includes endotheliology, endotheliopathy, and endotheliotherapy.

[Work supported by grants from NIH-USA, European Community, Romanian Academy and Ministry of Education and Research, Romania]

**A 14 — Arrhythmogenic right ventricular cardiomyopathy; a clinical and genetic perspective on desmosomes, desmin and calcium**

J. Peter van Tintelen

Department of (Clinical) Genetics, University Medical Center Groningen, Groningen, The Netherlands

p.van.tintelen@medgen.umcg.nl

Arrhythmogenic right ventricular cardiomyopathy (ARVC) is an inherited cardiomyopathy which usually presents with palpitations or syncope due to ventricular tachyarrhythmias. These arrhythmias may also underlie sudden death at relatively young ages. The general hallmark of this disease is (fibro-)fatty replacement of myocardial tissue, which contributes to activation delay, reentry and subsequent ventricular tachycardia. The areas of fibrofatty replacement are mainly localized in the right ventricle (RV).

The diagnosis of ARVC is made using generally accepted task force criteria. The initial criteria published in 1994 excluded left ventricular (LV) involvement. However, in recent years it has become clear that serious LV involvement can be found in ARVC as well. Recently, more left-dominant forms of "ARVC" were observed (ALVC). Based on these observations, the task force criteria for ARVC were recently modified to improve diagnostic sensitivity while maintaining specificity. Therefore, quantitative criteria were proposed. Besides, LV involvement is no longer an exclusion criterion, while the identification of a pathogenic mutation for ARVC is incorporated in the new criteria.

The elucidation of genes underlying ARVC started in 2000 with the discovery that autosomal recessive Naxos disease — a cardiocutaneous syndrome with ARVC and skin and hair abnormalities — was due to a deletion in the gene encoding plakoglobin. This is an important part of the desmosome. This discovery paved the road for the discovery of genes underlying autosomal dominantly inherited ARVC, such as plakophilin 2 (*PKP2*), desmoglein 2, desmocollin 2, and desmoplakin, all encoding proteins of the cardiac desmosome. Recently, desmin was added to this list. Because of the clinical overlap between left sided "ARVC" and dilated cardiomyopathy (DCM) a disease mainly involving the LV, we recently screened the phospholamban gene (*PLN*), a calcium-handling gene, in large DCM and ARVC cohorts from the Netherlands. This led to the identification of a previously described *PLN* mutation (R14del) in 10–15% of patients in both groups. This mutation has previously been described in DCM patients from other countries, and preliminary data show that this might be a global founder mutation. Interestingly, myocardial biopsies in *PLN* R14del ARVC patients show absent or reduced plakoglobin at the intercalated disk which has been suggested to be specific for ARVC, while the *PLN* R14del DCM patients generally show normal amounts of plakoglobin at the intercalated disk. These observations shift the paradigm that ARVC is solely due to mutations in desmosomal genes. The majority (>40 %) of ARVC patients carry mutations in *PKP2*. If we consider proven familial cases of ARVC, *PKP2* mutations can be identified in up to 70 % of families. This might suggest highly penetrant disease; however, not all desmosomal gene mutation carriers develop disease. This is also reflected by some recent large-scale population-based studies from Finland and the United States. In these studies, 0.5 % of healthy controls carried pathogenic mutations predisposing to ARVC, while the prevalence of ARVC is believed to be 1:1000–1:5000. The non-penetrance, clinical variability, and high prevalence of mutations in the general population hampers tailor-made therapy and surveillance in mutation carriers, who have a chance of dying suddenly, even in the absence of abnormalities in cardiological evaluation.

**A 15 — The changing spectrum of arrhythmogenic cardiomyopathy**

Cristina Basso

Department of Medico-Diagnostic Sciences and Special Therapies, University of Padua Medical School, Padua, Italy

cristina.basso@unipd.it

Arrhythmogenic cardiomyopathy (AC) is a clinically and genetically heterogeneous heart muscle disorder associated with ventricular arrhythmias and even risk of sudden death. The disease is heredo-familial, and mutations in desmosomal genes (plakoglobin, desmoplakin, plakophilin-2, desmoglein-2, and desmocollin-2) have been identified in up to 70 % of affected probands. Recent experimental models confirm that this genetically determined cardiomyopathy develops after birth due to progressive myocardial dystrophy. There is no single diagnostic "gold standard", and in 2010 a revision of the diagnostic criteria, encompassing familial, electrocardiographic, arrhythmic, morphofunctional, and histopathologic findings, was made to improve diagnostic sensitivity, but maintaining diagnostic specificity. Quantitative parameters for imaging, tissue characterization, ECG, and signal averaged ECG have been introduced, and the identification of a pathogenic mutation in a first degree relative has become a major criterion. Genotype–phenotype correlations, including magnetic resonance and pathology studies on heart specimens coming from heart transplant or sudden death, are currently demonstrating that the spectrum of the disease, usually referred to with the adjective "right ventricular", is wider than initially thought, with the evidence of biventricular or even isolated left ventricular forms, so that it is increasingly identified simply as "*arrhythmogenic cardiomyopathy*". Experimental cellular and animal models are mandatory to try to gain an insight into the cascade of cellular and molecular events leading from gene defect to myocardial dystrophy in AC.

**A 16 — Disease mechanisms in arrhythmogenic cardiomyopathy: immunohistochemistry and beyond**

Jeffrey E. Saffitz

Beth Israel Deaconess Medical Center/Harvard Medical School, Boston, MA, USA

jsaffitz@bidmc.harvard.edu

Arrhythmogenic right ventricular cardiomyopathy (ARVC) is a primary myocardial disorder characterized by an especially high incidence of ventricular arrhythmias and sudden death. The identification of desmosomal gene mutations in ∼50 % of patients has led to the idea that ARVC is a disease of abnormal cell–cell adhesion. However, recent insights gained largely from studies of the human disease suggest a greater degree of complexity. These insights include observations that: (1) plakoglobin (γ-catenin) is consistently redistributed from desmosomes to intracellular/nuclear sites in all forms of ARVC regardless of the underlying mutation, (2) ARVC patients have elevated circulating cytokine levels and myocardial production of cytokines, and (3) disease flares often follow strenuous exercise in ARVC patients. These and other observations have helped us to propose an overarching working model of the disease pathway in ARVC. A central feature of this model is that desmosomal mutations in ARVC result in abnormal responses to mechanical load which, in turn, promote subcellular redistribution of plakoglobin and induce cytokine expression. Redistribution of plakoglobin from junctional to intracellular/nuclear pools sets the stage for altered canonical and non-canonical Wnt signaling pathways with potential changes in gene expression and perturbation of Ca^2+^ homeostasis. Cytokines locally expressed by injured cardiac myocytes may promote development of fibrogenic and adipogenic phenotypes in the myocardium through paracrine effects on resident fibroblasts and progenitor cells. Altered cellular biomechanics downstream of ARVC-causing mutations also promote gap junction remodeling and cardiac myocyte apoptosis. Collectively, these alterations lead to cell injury, promote arrhythmogenesis, and contribute to the pathology of the disease. We now have multiple lines of evidence from studies of human disease and experimental systems to support major aspects of this disease model and to inform future studies. The hope is that insights gained about mechanisms of cell injury and arrhythmogenesis in ARVC may apply to more common forms of heart disease and sudden cardiac death.

**A 17 — Complex genetic behavior in arrhythmogenic cardiomyopathy and the effect on disease mechanisms**

Jeffrey A. Towbin

The Heart Institute and The Kindervelt–Samuel Kaplan Professor and Chief, Pediatric Cardiology, Cincinnati Children´s Hospital Medical Center, Cincinnati, OH, USA

jeffrey.towbin@cchmc.org

Objective: to define the genetic and mechanistic basis of arrhythmogenic cardiomyopathy. Background: arrhythmogenic cardiomyopathy, previously known as arrhythmogenic right ventricular dysplasia (ARVD) or arrhythmogenic right ventricular cardiomyopathy (ARVC), is characterized by myocardial fibrofatty replacement, ventricular dysfunction, and arrhythmias associated with sudden death. Autosomal dominant inheritance is most common, and reduced penetrance is the rule of thumb, but the underlying basis of low penetrance is poorly understood. The causative genes identified to date are generally associated with desmosome function, the "final common pathway" of this disease. We previously described compound and digenic heterozygosity as a feature of this disorder which plays a role in the development of the clinical phenotype and the severity of disease. We have continued to perform genetic screening of 275 probands and families with arrhythmogenic cardiomyopathy, and have identified mutations in desmosome-encoding genes, as well as in genes that are not primarily involved in the desmosome but disrupt the desmosome when mutated in at-risk domains. In addition, we have developed animal models of several of these genes, and these models are providing insight into the biology of the phenotype. These genes will be discussed, and the animal models developed will also be described, along with data on potential mechanisms of disease. Together, the data presented will support the concepts that: (1) the complex genetic basis of arrhythmogenic cardiomyopathy includes reduced penetrance with compound and digenic heterozygosity, (2) genes encoding non-desmosomal proteins result in arrhythmogenic cardiomyopathy by disrupting desmosome function by abnormal protein–protein interactions, and (3) disturbed junctional cytoarchitecture in subjects and animals with gene mutations that alter the desmosome confirms that arrhythmogenic cardiomyopathy is a disease of the desmosome and cell junctions.

*[Note: This topic was not orally presented because the invited speaker could not attend the workshop for health reasons]*

**A 18 — Naxos cardiocutaneous syndrome: recessive and dominant forms**

Adalena Tsatsopoulou, Nikos Protonotarios

Yannis Protonotarios Medical Center, Hora Naxos, 84300, Greece

adalena@otenet.gr

Naxos cardiocutaneous syndrome associates arrhythmogenic cardiomyopathy with hair and skin abnormalities. The original description of the syndrome has been reported in 1986 for families originating from the Aegean island of Naxos as a recessively inherited association of woolly hair, palmoplantar keratoderma and arrhythmogenic right ventricular cardiomyopathy, given the name of "Naxos disease".

The disease phenotype initially presents with woolly hair from infancy developing palmoplantar hyperkeratosis along with the increasing use of hands and feet. The cardiomyopathy becomes clinically apparent during adolescence or young adulthood, with ventricular arrhythmias, depolarization/repolarization changes in resting ECG, and structural/functional abnormalities of the right as well as the left ventricle, detected on two-dimensional echocardiography and cardiac magnetic resonance. Symptomatic presentation is with syncope, episodes of sustained ventricular tachycardia, or even sudden death. Heart failure might appear at the end stages of the disease, or earlier in cases with severe or predominant left ventricular involvement, as in the Carvajal variety of the Naxos cardiocutaneous syndrome. Cardiac pathology reveals myocyte loss and fibrous or fibrofatty replacement of myocardium, involving mostly subepicardial and mediomural layers. Up to now, families showing members with Naxos disease have also been identified on other Aegean islands. Moreover, cases from India, Ecuador, Italy, Israel, Saudi Arabia, Turkey, United Kingdom, Spain, and Finland have been reported with increasing frequency. They mostly appear with autosomal recessive inheritance, but autosomal dominant cases have also been described.

A 2-base-pair deletion mutation in plakoglobin, a key protein involved in cell–cell adhesion and signaling, was the first identified gene to cause Naxos cardiocutaneous syndrome, and indicated an involvement of deficient cell–cell adhesion in the pathogenesis of arrhythmogenic cardiomyopathy. Subsequently, mutations in the desmosomal plaque protein desmoplakin (DSP) and recently in the transmembrane glycoprotein desmocollin-2 have been found to be associated with recessive forms of Naxos cardiocutaneous syndrome, while dominant or digenic DSP mutations have been also related to the syndrome. The clinical appearance of cardiomyopathy with respect to the predominance of right or left ventricular involvement seems to be related to the site of cell junction plaque where the causative mutation is effective, i.e., mutations of desmoplakin truncating the desmin-binding site of the molecule lead either to predominant left ventricular involvement or to left dominant arrhythmogenic cardiomyopathy overlapping clinically with dilated cardiomyopathy (Carvajal syndrome). Dominant forms usually show milder palmoplantar keratoderma, while woolly hair is a constant finding in either dominant or recessive cases. The associated skin defects apparent from early infancy on enable the identification of individuals with concealed heart disease when arrhythmogenic cardiomyopathy is in an "incubation" stage. Thus, a clinical "electrocardiographic/arrhythmic" phase can often be documented to precede structural cardiomyopathy changes; molecular pathology at this stage has also indicated remodeling of gap junctions and electrical conduction.

**A 19 — Inherited arrhythmia syndromes: clinical and genetic management**

Michael H. Gollob

Inherited Arrhythmia Clinic, Division of Cardiology and Department of Cellular and Molecular Medicine, University of Ottawa Heart Institute, Ottawa, Ontario, Canada

mgollob@ottawaheart.ca

Arrhythmogenic right ventricular cardiomyopathy (ARVC), with an estimated prevalence of ARVC 1 per 5,000, is characterized by fibrofatty replacement of myocardium. It affects the right ventricle predominantly but may have left ventricular involvement. ARVC can result in ventricular arrhythmias, SCD, and right or biventricular dysfunction. Often sporadic, the condition is familial in up to 50 % of index cases. A pathogenetic theme for ARVC is the presence of mutations in genes encoding desmosomal proteins. Desmosomes are a primary component of cell-adhesion junctions, ensuring the structural and functional integrity of cardiomyocytes. Mutations have been identified in genes encoding for desmosomal proteins plakophilin-2 (*PKP2*), desmoplakin (*DSP*), plakoglobin (*JUP*), desmocollin (*DSC2*), and desmoglein (*DSG2*). A mutation in the gene encoding a nondesmosomal protein (*TMEM43*) has been identified as the cause in a large cohort of related patients in Newfoundland, Canada. The cellular function of the tmem43 protein is still uncertain. The epidemiological situation of the "Newfoundland cluster" and the health management consequences are disucssed. In addition, the involvement of cardiac gap junctions in atrial fibrillations is emphasized.

**A 20 — Carvajal/Naxos syndrome secondary to desmoplakin-dominant mutation is associated with dilated cardiomyopathy, woolly hair, palmoplantar keratoderma and hypo/oligodontia**

Lara Chalabreysse^1,2,5^, Faiza Senni^1,5^, Patrick Bruyère^3^, Brigitte Aime^1^, Christophe Ollagnier^1^, André Bozio^4^, Patrice Bouvagnet ^1,4,5^

(1) Laboratoire Cardiogénétique, (2) Service de Pathologie, (3) Service de Chirurgie Maxillo-faciale et Stomatologie, (4) Service Cardiologie Pédiatrique, Hôpitaux de Lyon, Lyon, France (5) Université de Lyon, F-69008 Lyon, France

patrice.bouvagnet@chu-lyon.fr

There is evidence that Carvajal (woolly hair, palmoplantar keratoderma and dilated cardiomyopathy) and Naxos (same hair and skin anomalies, with fibrofatty cardiomyocyte replacement in the right ventricle) syndromes are variable expressions of the same syndrome secondary to mutations in genes encoding proteins of the desmosome. Here we report an additional sign that might be helpful to cardiologists in establishing the diagnosis. The proband had three episodes (at age 15, 16 and 27) of chest pain with transient ST elevation in leads V3–V4 and mild rise of troponin. He had LV enlargement (diastolic diameter 64 mm) with normokinetics and no coronary anomalies. He had good adaptation to physical activity, but numerous premature beats disappearing during physical stress. He had palmoplantar keratoderma, woolly hair, and was missing the left mandibular 2nd molar and all 3rd molars. A younger brother had two fainting episodes when he was 17. He had incomplete RBBB and dilated left ventricle with normokinetics. Four years later, he had shortness of breath with LV at 70 mm and EF at 20 %. He had runs of ventricular tachycardia. He received a heart graft. His heart had enlarged ventricles with fibrofatty replacement in the right ventricle. He had palmoplantar keratoderma, woolly hair, and marked oligodontia with only four permanent molars and several persisting primary teeth. The father, who experienced several fainting spells, had also a dilated cardiomyopathy with the same skin, hair, and teeth anomalies. The desmoplakin (DSP) and plakoglobin (JUP) genes were screened for mutations, and a single heterozygous mutation was found in the DSP gene: p.Ser597Leu. This residue is conserved across vertebrates and absent from 100 controls. The mutation was found in the two brothers and their father, but absent from all other family members. Conclusion: heterozygous missense mutation in the DSP gene may result in chest pain, fainting episodes, and dilated cardiomyopathy. The association of woolly hair, palmoplantar keratoderma, and/or oligodontia may help in establishing the diagnosis of Carvajal/Naxos disease.

**A 21 — Reduction of preload: a pathophysiological approach to therapy in arrhythmogenic right ventricular cardiomyopathy?**

Paulus Kirchhof

Birmingham University, UK, and University Hospital Münster, Germany

KirchhP@ukmuenster.de

The observations and thoughts reported here have been published in JACC (1).

Arrhythmogenic right ventricular cardiomyopathy (ARVC) is an important cause of sudden death in the young (2–6). Despite progress in the prevention of sudden cardiac death in ARVC patients by antiarrhythmic drugs and implanted defibrillators (7–8), there is no treatment available that slows or prevents development of ARVC (2, 9–10).

Mutations in genes encoding for mechanical cell–cell contact proteins are found in ARVC patients (11–17). Transgenic mouse models have verified the biological relevance of mechanical cell–cell contact dysfunction for ARVC. The disease develops in mice with heterozygous deletion of plakoglobin (18), heterozygous deletion of desmoplakin (19), and transgenic expression of desmoglein (20). Recent analyses in myocardial tissue from ARVC patients suggest that reduced immuno-histological plakoglobin light intensity may be a sensitive and specific marker for ARVC in patients (5). Hence, genetic defects in the mechanical cell–cell contacts appear to be the underlying pathophysiological principle of ARVC. Endurance training, especially when performed at a competitive level, may accelerate the manifestation of ARVC in susceptible individuals (21–22). Likewise, endurance training accelerates the development of ARVC in heterozygous plakoglobin-deficient mice (plako^+/-^, [18]), suggesting that chronically increased volume load may contribute to development of ARVC in susceptible patients. We therefore tested whether reducing ventricular pressure and volume load can prevent or slow training-induced development of ARVC.

**Objective:** We used a murine model of arrhythmogenic right ventricular cardiomyopathy (ARVC) to test whether reducing ventricular load prevents or slows development of this cardiomyopathy.

**Methods:** Littermate pairs of heterozygous plakoglobin-deficient mice (plako^+/-^) and wild-type (WT) littermates underwent 7 weeks of endurance training (daily swimming). Mice were randomized to blinded load-reducing therapy (furosemide and nitrates) or placebo.

**Results:** Therapy prevented training-induced right ventricular (RV) enlargement in plako^+/-^ mice (RV volume: untreated plako^+/-^ 136±5μl; treated plako^+/-^ 78 ± 5 μl; WT 81 ± 5 μl; *p* < 0.01 for untreated vs WT and untreated vs treated; mean ± SEM). In isolated, Langendorff-perfused hearts, ventricular tachycardias (VT) were more often induced in untreated plako^+/-^ hearts (15/25) than in treated plako^+/-^ hearts (5/19) or in wild-type hearts (6/21, both *p* < 0.05). VT occurred due to macro-reentry and decreased RV longitudinal conduction velocity in untreated but not in treated plako^+/-^ mice (*p* < 0.01 for untreated vs WT and untreated vs treated). Myocardial concentration of phosphorylated connexin43 was lower in plako^+/-^ hearts with VTs compared to hearts without VTs, and was reduced in untreated plako^+/-^ compared to WT (both *p* < 0.05). Plako^+/-^ hearts showed reduced myocardial plakoglobin concentration, while β-catenin and N-cadherin concentration was not changed.

**Conclusions:** Load-reducing therapy prevents development of ARVC in plako^+/-^ mice, and may provide a simple approach to preventing disease progression in ARVC patients. This potential effect of preload reduction should be tested in a controlled clinical trial.

1. Fabritz L, Hoogendijk M, Scicluna BP, Van Amersfoorth SC, Fortmueller L, Wolf S et al (2011) Preload-reducing therapy prevents expression of arrhythmogenic right ventricular cardiomyopathy in plakoglobin-deficient mice. J Am Coll Cardiol 57:740–750

2. Marcus FI, McKenna WJ, Sherrill D, Basso C, Bauce B, Bluemke DA et al (2010) Diagnosis of arrhythmogenic right ventricular cardiomyopathy/dysplasia: proposed modification of the task force criteria. Circulation 121(13):1533–1541

3. Corrado D, Basso C, Pavei A, Michieli P, Schiavon M, Thiene G (2006) Trends in sudden cardiovascular death in young competitive athletes after implementation of a preparticipation screening program. Jama 296(13):1593–1601

4. Maron BJ, Towbin JA, Thiene G, Antzelevitch C, Corrado D, Arnett D et al (2006) Contemporary definitions and classification of the cardiomyopathies: an American Heart Association Scientific Statement from the Council on Clinical Cardiology, Heart Failure and Transplantation Committee; Quality of Care and Outcomes Research and Functional Genomics and Translational Biology Interdisciplinary Working Groups; and Council on Epidemiology and Prevention. Circulation 113(14):1807–1816

5. Asimaki A, Tandri H, Huang H, Halushka MK, Gautam S, Basso C et al (2009) A new diagnostic test for arrhythmogenic right ventricular cardiomyopathy. N Engl J Med 360(11):1075–1084

6. Maron BJ (2003) Sudden death in young athletes. N Engl J Med 349(11):1064–1075

7. Wichter T, Paul M, Wollmann C, Acil T, Gerdes P, Ashraf O et al (2004) Implantable cardioverter/defibrillator therapy in arrhythmogenic right ventricular cardiomyopathy: single-center experience of long-term follow-up and complications in 60 patients. Circulation 109(12):1503–1508

8. Wichter T, Borggrefe M, Haverkamp W, Chen X, Breithardt G (1992) Efficacy of antiarrhythmic drugs in patients with arrhythmogenic right ventricular disease. Results in patients with inducible and noninducible ventricular tachycardia. Circulation 86(1):29–37

9. Corrado D, Thiene G (2006) Arrhythmogenic right ventricular cardiomyopathy/dysplasia: clinical impact of molecular genetic studies. Circulation 113(13):1634–1637

10. Thiene G, Basso C, Calabrese F, Angelini A, Valente M (2005) Twenty years of progress and beckoning frontiers in cardiovascular pathology: cardiomyopathies. Cardiovasc Pathol 14(4):165–169

11. Yang Z, Bowles NE, Scherer SE, Taylor MD, Kearney DL, Ge S et al (2006) Desmosomal dysfunction due to mutations in desmoplakin causes arrhythmogenic right ventricular dysplasia/cardiomyopathy. Circ Res 99(6):646–655

12. Gerull B, Heuser A, Wichter T, Paul M, Basson CT, McDermott DA et al (2004) Mutations in the desmosomal protein plakophilin-2 are common in arrhythmogenic right ventricular cardiomyopathy. Nat Genet 36(11):1162–1164

13. McKoy G, Protonotarios N, Crosby A, Tsatsopoulou A, Anastasakis A, Coonar A et al (2000) Identification of a deletion in plakoglobin in arrhythmogenic right ventricular cardiomyopathy with palmoplantar keratoderma and woolly hair (Naxos disease). Lancet 355(9221):2119–2124

14. Pilichou K, Nava A, Basso C, Beffagna G, Bauce B, Lorenzon A et al (2006) Mutations in desmoglein-2 gene are associated with arrhythmogenic right ventricular cardiomyopathy. Circulation 113(9):1171–1179

15. Syrris P, Ward D, Asimaki A, Sen-Chowdhry S, Ebrahim HY, Evans A et al (2006) Clinical expression of plakophilin-2 mutations in familial arrhythmogenic right ventricular cardiomyopathy. Circulation 113(3):356–364

16. van Tintelen JP, Entius MM, Bhuiyan ZA, Jongbloed R, Wiesfeld AC, Wilde AA et al (2006) Plakophilin-2 mutations are the major determinant of familial arrhythmogenic right ventricular dysplasia/cardiomyopathy. Circulation 113(13):1650–1658

17. Heuser A, Plovie ER, Ellinor PT, Grossmann KS, Shin JT, Wichter T et al (2006) Mutant desmocollin-2 causes arrhythmogenic right ventricular cardiomyopathy. Am J Hum Genet 79(6):1081–1088

18. Kirchhof P, Fabritz L, Zwiener M, Witt H, Schafers M, Zellerhoff S et al (2006) Age- and training-dependent development of arrhythmogenic right ventricular cardiomyopathy in heterozygous plakoglobin-deficient mice. Circulation 114(17):1799–1806

19. Garcia-Gras E, Lombardi R, Giocondo MJ, Willerson JT, Schneider MD, Khoury DS et al (2006) Suppression of canonical Wnt/beta-catenin signaling by nuclear plakoglobin recapitulates phenotype of arrhythmogenic right ventricular cardiomyopathy. J Clin Invest 116(7):2012–2021

20. Pilichou K, Remme CA, Basso C, Campian ME, Rizzo S, Barnett P et al (2009) Myocyte necrosis underlies progressive myocardial dystrophy in mouse dsg2-related arrhythmogenic right ventricular cardiomyopathy. J Exp Med 206(8):1787–1802

21. Heidbuchel H, Hoogsteen J, Fagard R, Vanhees L, Ector H, Willems R et al (2003) High prevalence of right ventricular involvement in endurance athletes with ventricular arrhythmias. Role of an electrophysiologic study in risk stratification. Eur Heart J 24(16):1473–1480

22. Maron BJ, Chaitman BR, Ackerman MJ, Bayes de Luna A, Corrado D, Crosson JE et al (2004) Recommendations for physical activity and recreational sports participation for young patients with genetic cardiovascular diseases. Circulation 109(22):2807–2816

**A 22 — Sudden death in athletes**

Gaetano Thiene

Department of Medico-Diagnostic Sciences and Special Therapies, University of Padua Medical School, Padua, Italy

gaetano.thiene@sanita.padova.it

Sudden death in athletes occurs due to hidden cardiac disorders which, often during effort, may jeopardize heart electrical stability, triggering ventricular fibrillation. Apart from rare conditions of ion channel diseases in a structurally normal heart, in which the disorder may be detected simply by basal or stress test 12 lead ECG, the anomalies at risk of sudden death may affect the aorta (Marfan syndrome), the coronary arteries (congenital anomalies, premature atherosclerosis), the myocardium (hypertrophic and arrhythmogenic cardiomyopathies), the valves (bicuspid aortic valve, mitral valve prolapse) and the conduction system (preexcitation syndromes). These disorders are for the most part detectable by ECG and/or echo. The employment of these methods in screening tests before competitions or other athletic meetings can help to identify masked anomalies and should play a major role in early diagnosis, risk stratification and prevention of sudden death.

**A 23 — Wnt-Ca** ^**2+**^ **as an integrator of differentiation signaling in the heart**

Calum A. MacRae

Cardiovascular Division, Brigham & Women´s Hospital, Boston, MA, USA

camacrae@bics.bwh.harvard.edu

Intercellular junctions have been directly implicated in the pathobiology of several forms of human cardiomyopathy through human genetic studies. The downstream mechanisms of the junctional perturbations remain obscure, but to date abnormalities of mechanical and electrochemical signaling have been identified. We have used the zebrafish to model multiple aspects of junctional biology during normal and pathological cardiogenesis.

In the first instance, we focused on developing techniques to characterize the dynamics of intercellular coupling during normal cardiac development. High-resolution optical voltage mapping, Fura-2 calcium imaging and traditional immunohistochemical techniques were combined to define cardiomyocyte differentiation and cell coupling at multiple stages from the linear heart tube stage through to 120hpf, when the zebrafish heart is highly representative of adult human cardiac physiology. The structural and functional remodeling of junctions is coupled with cytoskeletal and membrane lipid remodeling which we are also beginning to characterize. We have identified remarkable plasticity of cardiomyocyte coupling throughout development, with distinctive regional variation in the complexity and strength of coupling. These data demonstrate that the myocardial syncytium is not uniform, but rather consists of a complex series of local networks. Exploration of a series of zebrafish mutants has identified roles for pathways implicated in synaptogenesis in the formation of specific regional coupling networks.

Interestingly, non-canonical Wnt signals polarize ventricular myocardial structure and function, but do not perturb the major ventricular cardiomyocyte networks. We were unable to detect any role for traditional planar cell polarity pathways in the Wnt-associated polarity changes, but identified a specific role for the L-type calcium channel in these cellular and physiologic rearrangements. This novel limb of Wnt–calcium signaling directly connects extracellular and intracellular compartments, and may serve to integrate canonical and non-canonical Wnt pathways.

Given the role of desmosomal proteins in human cardiomyopathy, we established and validated multiple zebrafish lines expressing dominant negative mutants implicated in disease. Using a range of reporter lines, and existing zebrafish Wnt signaling mutants, there is evidence for both canonical and non-canonical Wnt pathway abnormalities in desmosomal cardiomyopathies. We have used one of our lines expressing a mutant plakoglobin line to develop a high-throughput screen for genetic and chemical suppressors of desmosomal pathology. This line develops a cardiomyopathy with extensive morphologic and physiologic homology to ARVC, and is associated with reduced survival, sudden death and congestive heart failure. We are currently evaluating novel Wnt–calcium pathway members identified in zebrafish, and screening chemical libraries for novel modulators of desmosomal disease pathways for use as pathway probes, diagnostics or therapeutic leads.

**A 24 — Clinical challenges in ARVC/D: a United States perspective**

Hugh M. Calkins

Division of Cardiology, Department of Medicine, Johns Hopkins University School of Medicine, Baltimore, MD, USA

hcalkins@jhmi.edu

The Johns Hopkins ARVD Program was initiated in 1999 with the goal of furthering research on ARVD and also providing clinical care for patients with known or suspected ARVD. During the past 10 years, we have evaluated more than 1,000 patients for this condition. The purpose of this presentation will be to present our clinical experience with ARVD and to focus on what we perceive to be the more vital unanswered questions. Particular emphasis will be placed on describing the remarkable heterogeneity of the clinical manifestations of the disease. Our aim will be to provide a clinical context for the remarkable advances in basic research that will be discussed at this meeting.

**A 25 — The molecular biology and genetics of blood pressure and its regulation**

John J. Mullins

The Centre for Cardiovascular Science, The University of Edinburgh, Queen´s Medical Research Institute, Edinburgh, UK

j.mullins@ed.ac.uk

Mammalian blood pressure is regulated by a myriad of factors, many of which have been identified by classical biochemical and physiology experimentation, whilst others have emerged through genetic studies in both human populations and rodent models. The advent of genetic manipulation in vivo has facilitated the investigation of mechanisms underlying inherited forms of hypertension and the cell biology and physiology of altered blood pressure homeostasis.

Renin is a critical component of a classical renin–angiotensin–aldosterone system (RAAS), which regulates long-term mammalian blood pressure. We have shown that a threshold level of expression of the renin is an essential determinant of the morphology and function of the renal juxtaglomerular cells and of the structure of the anatomically and functionally related macula densa cells. In genetic rescue experiments, we have shown that human renin expression in mice lacking endogenous renin1 enzyme rescues the JG cell deficit but is not sufficient, in isolation, to restore normal macula densa structure.

Two of the key effectors of the classic RAAS are angiotensin II (Ang II) and aldosterone. Whilst much attention has been paid to the actions of Ang II in regulating blood pressure, the dysregulation of the aldosterone axis is also known to lead to profound physiological and pathophysiological consequences, including a marked elevation of blood pressure. Aldosterone is not the sole activator of MR in vivo, and glucocoticoids are known to bind to MR with similar efficiency to that of aldosterone. The relative abundance of glucocorticoids in the plasma provides the potential for substantive glucocorticoid-mediated activation of MR. Under normal circumstances, such activation is prevented by the enzyme 11 beta hydroxysterioid dehydogenase type 2 (11 beta HSDII), which acts locally to inactivate glucocorticoids and thereby prevents their access to MR, resulting in the apparent specificity of aldosterone action. Lack of 11 beta HSDII activity in humans results in the syndrome of apparent mineralocorticoid excess (SAME), a severe hypertensive disorder with characteristic electrolyte imbalance and a poor prognosis. We have established a transgenic mouse model of SAME which recapitulates all the key features of the human condition and facilitates investigation of the underlying mechanism of the syndrome and the consequences of chronic reduction in the expression of this critical "gate-keeper" enzyme. Current developments and potential pitfalls of the use of genetic modification will be discussed, with a focus on its use in investigating blood pressure homeostasis.

**A 26 — The specific molecular ensembles of the junctions connecting mesenchymally-derived tumor cells, including cardiac myxomata: the coming and going of plakophilin-2**

Steffen Rickelt ^1,2^, Stefania Rizzo^3^, Mareike Barth^1,4^, Werner W. Franke^1,2^

(1) Helmholtz Group for Cell Biology, German Cancer Research Center (DKFZ), Heidelberg, Germany

(2) Progen Biotechnik, Heidelberg, Germany

(3) Department of Medical-Diagnostic Sciences, University of Padua Medical School, Padua, Italy

(4) Institute for Pharmacology and Clinical Pharmacology, University Hospital Düsseldorf, Düsseldorf, Germany

s.rickelt@dkfz-heidelberg.de

In contrast to the advanced state of the molecular characterization of the junctions connecting epithelial cells, i.e., the intermediate-sized filament (IF)-anchoring desmosomes (maculae adhaerentes) and the microfilament-associated adherens junctions (AJs), knowledge of the molecular components of the AJs that connect the diverse types of mesenchymally-derived cells — normal or malignantly transformed — is still very limited. Therefore, we have begun to study the molecular composition and the assembly mechanisms of the AJs connecting the cells of mesenchymally-derived tissues and soft-tissue tumors. In a systematic study to elucidate the molecular composition of the AJs that connect non-epithelial cells, we have identified a series of novel and frequent cell type-specific molecular AJ ensembles, some of which represent unexpectedly new categories of junctional assemblies, including molecules hitherto only known as major components of desmosomes in epithelial and carcinomatous cells. Using biochemical and immunocytochemical as well as electron microscopical methods, we have identified a category of AJs connecting diverse normal and proliferatively-transformed, mesenchymally-derived cells, which in addition to N-cadherin and/or cadherin-11 contain a dense cytoplasmic plaque formed by a- and b-catenin, plakoglobin as well as proteins p120 and p0071, but also major amounts of the desmosomal plaque proteins plakophilins-2 (Pkp2) and/or -3 (Pkp3). Such mixed type AJs have been discovered in certain proliferatively active normal cell culture lines, as well as in malignantly transformed cells (Rickelt et al. 2009). In particular, we have found this novel AJ type in diverse kinds of human soft-tissue tumors, including rhabdomyosarcomas, and remarkably consistent in cardiac myxomata (without exception in all 38 cases examined so far; Rickelt et al. 2010). The specific correlation of the advent of such Pkp2-positive AJs with the proliferative state of the given cells is essentially well demonstrable with the interstitial cells of the cardiac valves (Barth et al. 2009). The possible diagnostic value of Pkp2 in tumor diagnosis is obvious.

Barth M, Schumacher H, Kuhn C, Akhyari P, Lichtenberg A, Franke WW (2009) Cordial connections: molecular ensembles and structures of adhering junctions connecting interstitial cells of cardiac valves in situ and in cell culture. Cell Tissue Res 337:63–77

Rickelt S, Rizzo S, Doerflinger Y, Zentgraf HW, Basso C, Gerosa G, Thiene G, Moll R, Franke WW (2010) A novel kind of tumor type characteristic junction: plakophilin-2 as a major protein of adherens junctions in cardiac myxomata. Mod Pathol 11:1429–1437

Rickelt S, Winter-Simanowski S, Noffz E, Kuhn C, Franke WW (2009) Upregulation of plakophilin-2 and its acquisition to adherens junctions identifies a novel molecular ensemble of cell–cell-attachment characteristic for transformed mesenchymal cells. Int J Cancer 125:2036–2048

**A 27 — N-cadherin antagonists as oncology therapeutics**

Orest W. Blaschuk

Division of Urology, Department of Surgery, McGill University, Montreal, Quebec, Canada

N-cadherin is a cell adhesion molecule (for review, see Hulpiau and van Roy 2008) that plays a pivotal role in promoting blood vessel formation and stability (for review, see Blaschuk and Devemy 2009). Both of these processes are essential for tumor growth (for review, see Folkman 2007; Naumov et al. 2008). Initially, I will discuss the discovery and development of N-cadherin antagonists. These antagonists consist of three series of compounds: linear peptides (Blaschuk et al. 1990), cyclic peptides (Williams et al. 2000) and peptidomimetics (Gour et al. 2007). In particular, I will focus on the effects of these antagonists on endothelial cell adhesion (Erez et al. 2004). Secondly, I will discuss pre-clinical (Shintani et al. 2008; Augustine et al. 2008) and clinical studies (Beasley et al. 2009; Perotti et al. 2009) designed to ascertain the effects of the N-cadherin antagonist, ADH-1 (a synthetic cyclic pentapeptide) on tumor growth. Collectively, the data suggest that N-cadherin antagonists might be useful as anti-cancer agents.

Augustine CK, Yoshimoto Y, Gupt M, Zipfel PA, Selim MA, Febbo P, Pendergast AM, Peters WP, Tyler DS (2008) Targeting N-cadherin enhances antitumor activity of cytotoxic therapies in melanoma treatment. Cancer Res 68:3777–3784

Beasley GM, McMahon N, Sanders G, Augustine CK, Selim MA, Peterson B, Norris R, Peters WP, Ross MI, Tyler DS (2009) A Phase 1 study of systemic ADH-1 in combination with melphalan via isolated limb infusion in patients with locally advanced in-transit malignant melanoma. Cancer 115:4766–4774

Blaschuk OW, Devemy E (2009) Cadherins as novel targets for anti-cancer therapy. Eur J Pharmacol 625:195–198

Blaschuk OW, Sullivan R, David S, Pouliot Y (1990) Identification of a cadherin cell adhesion recognition sequence. Develop Biol 139:227–229

Erez N, Zamir E, Gour BJ, Blaschuk OW, Geiger B (2004) Induction of apoptosis in cultured endothelial cells by a cadherin antagonist peptide: involvement of fibroblast growth factor receptor-mediated signalling. Exp Cell Res 294:366–378

Folkman J (2007) Angiogenesis: an organizing principle for drug discovery? Nat Rev Drug Discov 6:273–286

Gour BJ, Blaschuk OW, Ali A, Ni F, Chen Z, Michaud SD, Wang S, Hu Z (2007) Peptidomimetic modulators of cell adhesion. United States Patent Number 7,268,115

Hulpiau P, van Roy F (2008) Molecular evolution of the cadherin superfamily. Int J Biochem Cell Biol 41:349–369

Naumov GN, Folkman J, Straume O, Akslen LA (2008) Tumor–vascular interactions and tumor dormancy. APMIS 116:569–585

Perotti A, Sessa C, Mancuso A, Noberasco C, Cresta S, Locatelli A, Carcangiu ML, Passera K, Braghetti A, Scaramuzza D, Zanaboni F, Fasolo A, Capri G, Miani M, Peters WP, Gianni L (2009) Clinical and pharmacological phase I evaluation of Exherin (ADH-1), a selective anti-N-cadherin peptide in patients with N-cadherin-expressing solid tumours. Ann Oncol 20:741–745

Shintani Y, Fukumoto Y, Chaika N, Grandgenett PM, Hollingsworth MA, Wheelock MJ, Johnson KR (2008) ADH-1 suppresses N-cadherin-dependent pancreatic cancer progression. Int J Cancer 122:71–77

Williams E, Williams G, Gour BK, Blaschuk OW, Doherty P (2000) A novel family of cyclic peptide antagonists suggest that N-Cadherin specificity is determined by amino acids that flank the HAV motif. J Biol Chem 275:4007–4012

**A 28 — The cardiac fibroblast: phenotypes, functions and therapeutic target**

Karen E. Porter

Division of Cardiovascular & Neuronal Remodelling, School of Medicine, University of Leeds, UK

k.e.porter@leeds.ac.uk

Cardiac fibroblasts (CF) are the most prevalent cell type in the mammalian heart, yet until recent years their role in regulating myocardial function and remodelling has been largely overlooked in favour of the cardiac muscle cells, cardiomyocytes. The critical role of CF in maintaining myocardial homeostasis in the healthy heart is underscored by the need to provide structure, function, and connectivity for all the myocardial cell types. CF therefore play key roles in regulating not only normal myocardial function but also during adverse remodelling that occurs with hypertension, myocardial infarction (MI) and heart failure (HF). Through cell–cell interaction and secretion of growth factors, cardiomyocyte function is directly modulated by CF and vice versa. During the myocardial remodelling that follows MI and in HF progression, CF undergo activation to a myofibroblast phenotype, expressing contractile proteins such as α-SMA and modulating their function through increased proliferative, migratory, and secretory properties.

In the remodelling heart, CF are functionally responsive to the elevated levels of proinflammatory cytokines such as TNF-α and interleukins, vasoactive peptides (e.g., angiotensin II, endothelin-1) and hormones (e.g., noradrenaline). Such effects include changes in cell proliferation, cell migration, extracellular matrix turnover, and secretion of bioactive molecules including cytokines, peptides, and growth factors. Although these changes in function are an important adaptive response to myocardial injury, in the long term they lead to adverse remodelling and heart failure. Many commonly prescribed cardiovascular therapies (for example ACE inhibitors, beta-blockers, and statins) also exert pleiotropic effects on cardiac fibroblasts that may explain some of their benefits in the remodelling heart. In addition, as the importance of CF emerges and our understanding broadens, modulation of their phenotype and function may provide a worthwhile goal in the quest for novel, targeted therapeutics.

Mughal RS, Warburton P, O'Regan DJ, Ball SG, Turner NA, Porter KE (2009) Peroxisome proliferator-activated receptor γ-independent effects of thiazolidinediones on human cardiac myofibroblast function. Clin Exp Pharmacol Physiol 36:478–486

Porter KE, Turner NA (2009) Cardiac fibroblasts: at the heart of myocardial remodeling. Pharmacol Ther 123:255–278

Porter KE, Turner NA (2011) Statins and myocardial remodelling: cell and molecular pathways. Expert Rev Mol Med 13:e22

Porter KE, Turner NA, O'Regan DJ, Balmforth AJ, Ball SG (2004) Simvastatin reduces human atrial myofibroblast proliferation independently of cholesterol lowering via inhibition of RhoA. Cardiovasc Res 61:745–755

Turner NA (2011) Therapeutic strategies for targeting the cardiac fibroblast. In: Turner NA (ed) The cardiac fibroblast. Research Signpost, Kerala, pp 273–285

Turner NA, Porter KE, Smith WH, White HL, Ball SG, Balmforth AJ (2003) Chronic β_2_-adrenergic receptor stimulation increases proliferation of human cardiac fibroblasts via an autocrine mechanism. Cardiovasc Res 57:784–792

**A 29 — Cardiac myocyte–fibroblast electrical interactions**

Peter Kohl

Chair in Cardiac Biophysics and Systems Biology, National Heart and Lung Institute, London, UK

p.kohl@imperial.ac.uk

*"The heart is a muscle. Muscle is meat. Meat consists of myocytes. If you understand myocytes, you understand the heart."* If this was our conceptual approach to exploring cardiac structure–function relationships and electro-mechanical activity, we would be bound to fail. The heart contains more non-myocytes than muscle cells (Vliegen et al. 1991), and the majority of the former are referred to as fibroblasts (Adler et al. 1981). The traditional view of fibroblasts is dominated by regarding them as 'obstacles' (from blocking conduction in vivo, to overgrowing cell cultures in vitro). This perception is shifting, however, to give way to an increasingly integrative appreciation of the relevance of fibroblasts for proper function of cardiac myocytes (Camelliti et al. 2005). This trend is evident from increased publication numbers on the topic, as can be illustrated by a PubMed search for '(fibroblast) AND (heart OR cardiac)': out of 8,000+ entries returned for the term, more than half have been published in the past 10 years. As is evident from these numbers, cardiac myocyte–fibroblast interactions are a huge field. This presentation will focus, therefore, on a small aspect of those interactions: electrical cross-talk between the heterogeneous cell populations. A brief introduction of terms and concepts will be followed by a review of insight from 'wet' experimental and 'dry' computational basic research, a consideration of clinical correlates, an assessment of underlying molecular substrates for electrical cell–cell interaction, and an outlook that considers current technical and conceptual limitations, as well as possible solutions—regrettably, however, without answering the 'friend or foe?' question (Baudino et al. 2006).

Adler CP, Ringlage WP, Böhm N (1981) DNS-Gehalt und Zellzahl in Herz und Leber von Kindern. Pathol Res Pract 172:25–41

Baudino TA, Carver W, Giles W, Borg TK (2006) Cardiac fibroblasts: friend or foe? Am J Physiol (Heart Circ Physiol) 291:H1015–H1026

Camelliti P, Borg TK, Kohl P (2005) Structural and functional characterisation of cardiac fibroblasts. Cardiovasc Res 65:40–51

Vliegen HW, van der Laarse A, Cornelisse CJ, Eulderink F (1991) Myocardial changes in pressure overload-induced left ventricular hypertrophy: A study on tissue composition, polyploidization and multinucleation. Eur Heart J 12:488–494

**A 30 — Telocytes: a novel kind of mesenchymally derived cells in the heart and in other organs**

Lawrence M. Popescu ^1,2^

(1) Department of Cellular and Molecular Medicine “Carol Davila”, University of Medicine and Pharmacy, Bucharest, Romania

(2) Department of Advanced Studies, “Victor Babes” National Institute of Pathology, Bucharest, Romania

lpopescu@jcmm.org

We have recently described a novel type of interstitial cells — telocytes (TC) — in several cavitary and non-cavitary organs, from humans and mammals (Popescu and Faussone-Pellegrini 2010). TC were found in all the three concentric layers of heart: epi-, myo- and endocardium (see www.telocytes.com). Their presence was repeatedly confirmed and, moreover, a "map" of TC localization in heart was established (Liu et al. 2011). TC have a small body, but specific (unique) prolongations, that we named telopodes (Tp). Therefore, the simplest definition of telocytes (TC) is: cells with Tp. Tp are characterized by: (a) number (1–5, frequently 2–3), (b) length (several tens up to hundreds of micrometers), (c) moniliform aspect: alternation of dilated segments (podoms) and thin segments (podomers — less than 200 nm thickness, below the resolving power of light microscopy, explaining the fact that TC were overlooked so far), (d) podoms accommodating mitochondria, endoplasmic reticulum (ER) and caveolae — the so-called “Ca^2+^ uptake/release units”, and (e) dichotomous branching pattern, making a 3D network, a labyrinthine system with particular ultrastructural homo- and hetero-cellular junctions. It is worthy of note that TC and especially Tp release microvesicles (mean diameter of 180 nm), sending macromolecular signals to neigboring cells and eventually modifying their transcriptional activity.

The immunophenotype of TC includes mainly CD 34, CD117/c-Kit, and vimentin, but also caveolin-1, CD44, NOS-2, desmin, cadherin-11, PDGF-R beta and so on. Although for the time being, electron microscopy remains the election method to precisely identify TC, double-positive immunostaining with CD34/c-Kit (mainly for cell body) or CD34/vimentin (mainly for Tp) also represents a useful marker for TC. MicroRNA profile of TC is characterized by the absence of miRs 1, 133a, 208a, specific for cardiomyocytes, but the distinct presence of miR 608. Micro RNAs 126-3p, 151-5p, and 193 are differentially expressed by TC in comparison with other interstitial cells (e.g., fibroblasts), as resulted from laser capture microdissection. TC contain measurable quantities of angiogenic microRNAs (e.g., let-7e, 10a, 21, 27b, 100, 130a, 143, 155, 503) (Manole et al. 2011). It is worthy of mention that TC have a diverse paracrine secretion (VEGF, NO, IL6, chemokines), as suggested by immunohistochemistry and confirmed by SELDI-TOF mass spectrometry and xMAP technology (Luminex) for TC in tissue culture. In cell cultures, when compared to fibroblasts (3T3), TC produced 8 times more IL6 and 3 times more VEGF respectively. Transmission electron microscopy and electron tomography revealed complex junctions between TC and cardiomyocytes, as well as nanostructural junctions between TC and resident progenitor cells, at the level of epicardial stem cell niches (Gherghiceanu and Popescu 2010, 2011). It is worthy of note that close contacts between TC and putative progenitor cells were also found at the level of interstitial stem cell niches in lungs (Popescu et al. 2011), as well as in skeletal muscle (Popescu et al. 2011). TC, with their Tp, surround cardiac progenitors or precursors to guide them to form the coherent 3D myocardial architecture. Apparently, Tp provide “tracks” for the sliding of cardiomyocyte precursors in their development and integration as working cardiomyocytes.

In conclusion, in the adult mammalian heart, TC together with resident stem cells and cardiomyocyte progenitor sustain a continuous cardiac renewal process, and might be key players in repairing the damaged heart. TC “nurse” the progenitor cells in stem cell niches. The tandem TC–stem cells could be a better option for therapy rather than stem cells alone. Last but not least, TC are directly (physically) and indirectly (chemically) involved in neoangiogenesis after myocardial infarction.

Gherghiceanu M, Popescu LM (2010) Cardiomyocyte precursors and telocytes in epicardial stem cell niche: electron microscope images. J Cell Mol Med 14:871–877

Gherghiceanu M, Popescu LM (2011) Heterocellular communication in the heart: electron tomography of telocyte–myocyte junctions. J Cell Mol Med 15:1005–1011

Liu JJ, Shen XT, Zheng X, Li Z, Wang J, Q XF (2011) Distribution of telocytes in the rat heart. J Clin Rehabil Tissue Eng Res 15:3546–3548

Manole CG, Cismasiu V, Gherghiceanu M, Popescu LM (2011) Experimental acute myocardial infarction: telocytes involvment in neo-angiogenesis. J Cell Mol Med 15:2284–2296

Popescu LM, Faussone-Pellegrini MS (2010) Telocytes – a case of serendipity: the winding way from interstitial cells of Cajal (ICC), via interstitial Cajal-like cells (ICLC) to telocytes. J Cell Mol Med 14:729–740

Popescu LM, Gherghiceanu M, Suciu LC, Manole CG, Hinescu ME (2011) Telocytes and putative stem cells in the lungs: electron microscopy, electron tomography and laser scanning microscopy. Cell Tissue Res 345:391–403

Popescu LM, Manole E, Serboiu CS, Manole CG, Suciu LC, Gherghiceanu M., Popescu BO (2011) Identification of telocytes in skeletal muscle interstitium: implication for muscle regeneration. J Cell Mol Med 15:1379–1392

**A 31 — Periostin/filamin A: a candidate central regulatory mechanism for valvular fibrogenesis and matrix compaction**

Roger Markwald, Russell Norris, Suniti Misra, Shibnath Ghatak

Cardiovascular Developmental Biology Center, Department of Regenerative Medicine, Medical University of South Carolina, Charleston, SC, USA

markwald@musc.edu

Periostin (PN) is a fasciclin-related protein secreted by prevalvular primordial mesenchyme of the developing ventricular inlets and outlets. It is also expressed by interstitial cells of the embryonic and adult ventricular myocardium. We have previously shown that PN promotes cell autonomous, fibrogenic differentiation and the compaction of a collagenous matrix into mature valve leaflets and cusps. Loss of periostin inhibits differentiation into fibroblast lineages, collagen secretion and matrix compaction, resulting in abnormally elongated, myxomatous valves with potential for prolapse and regurgitation. With respect to mechanism(s), PN, as a matricellular protein, may promote matrix compaction and maturation by *directly* binding to collagen (thereby increasing cross-linking) or *indirectly* by binding to integrins and *potentially* initiating signaling changes in cytoskeleton organization that modify the contractile forces that mediate matrix compaction. To test the latter mechanism, we initiated signaling studies and a 3D assay to determine if PN promoted matrix compaction by activating integrin-associated signaling pathways, and if such activation included phosphorylation of A (FLNA), an actin binding, cytoskeletal protein specifically expressed in cardiac valve and interstitial fibroblasts. IP data, Western blots and immunostaining indicated that PN binding to 3 integrin specifically promoted phosphorylation of FLNA (a.a. 2152) by activating cdc42 and pak1 kinases, and that the 2152 phosphorylation site of FLNA occurred near two point mutations (P673**Q** and G228**R**) found in patients with degenerative (myxomatous) valve diseases. Silencing or deleting periostin inhibited 2152 FLNA phosphorylation, which correlated with reduced potential of prevalvular interstitial cells (also ventricular fibroblasts) to compact collagen gels. Introducing the P673Q and G228R mutations into mouse prevalvular mesenchyme inhibited both collagen compaction and the phosphorylation of FLNA (2152). Fibroblasts from patients with P673Q and G228R mutations or mouse fibroblasts expressing one or more of these mutations or silencing vectors for PN also exhibited reduced binding of an enzyme — transglutaminase 2 (TG2) — which covalently links serotonin to FLNA. Pharmacologically or genetically inhibiting "serotonylation" of FLNA strongly suppressed matrix compaction and valve maturation. Together, these findings indicate that PN can regulate valvulogenesis and matrix maturation by triggering integrin-dependent signaling mechanisms that target FLNA and its potential to generate cytoskeletal contractile forces through an interaction with TG2 and serotonin.

[Supported by NHLBI, AHA, NCRR and Leducq Mitral Transatlantic Network.]

**A 32 — Molecular mechanisms of heart valve development and disease**

Elaine E. Wirrig, Jonathan D. Cheek, Santanu Chakraborty, Christina M. Alfieri, Robert B. Hinton,

Katherine E. Yutzey

The Heart Institute, Cincinnati Children's Hospital Medical Center, Cincinnati, OH, USA

katherine.yutzey@cchmc.org

Studies of human explanted aortic valves and mouse models were used to define molecular, cellular, and morphogenetic features of heart valve disease progression. In human diseased aortic valves, valvular interstitial cell (VIC) activation, extracellular matrix (ECM) disorganization, and induction of markers of valve mesenchymal and cartilage progenitor cells are observed in both pediatric and adult diseased aortic valves. In contrast to calcific valve disease in adults, pediatric diseased aortic valves do not calcify, and expression of osteogenic markers is apparent in adult, but not pediatric, diseased aortic valves. Interestingly, phospho-Smad1/5/8 also is increased in adult calcified valves, suggesting that BMP signaling contributes to heart valve calcification. Osteogenesis imperfecta murine (Oim) mice have a mutation in Col1a2 that affects collagen fibrillogenesis and leads to bone fragility. The valves of Oim/Oim mice have decreased collagen and increased proteoglycan composition with leaflet thickening. In addition, the Oim/Oim mice have increased expression of valve and cartilage progenitor markers, consistent with increased proteoglycan composition and absence of calcification.

Klotho-null mice are a model for premature aging, and exhibit calcified nodules in aortic valves in the absence of leaflet thickening or ECM disorganization. Klotho-null aortic valves have increased expression of the transcription factor Runx2, consistent with the calcified phenotype, in addition to increased expression of cartilage marker genes. Together, these findings demonstrate specific molecular indicators of aortic valve disease progression, which could lead to identification of early disease markers and the development of therapeutic interventions.

**A 33 — Next generation sequencing in ARVC mutation detection and new genes for desmosomal-like disease**

David P. Kelsell

The Blizard Institute, Barts and The London School of Medicine and Dentistry, Queen Mary University of London, London, E1 4AT, UK

d.p.kelsell@qmul.ac.uk

The importance of desmosomes for maintenance of the strength and flexibility of these tissues is highlighted by natural and in vitro engineered mutations in desmosomal genes, which compromise skin or heart and in some instances both. Indeed, desmosomal gene mutations account for 45–50 % of cases of arrhythmogenic right ventricular cardiomyopathy (ARVC). ARVC is a disease of ventricular myocardium, which on macroscopic histological examination reveals ventricular dilatation, thinning of affected myocardium, and areas of scarring. Detailed genetic sequencing has now identified both heterozygous and mutations in all five major proteins within the desmosome. The current detection rate of pathogenic mutations in non-syndromic ARVC is 45–50 %, with mutations most commonly seen in PKP-2 and least frequently seen in PG. In this presentation, the role of targeted capture followed by next-generation sequencing in ARVC mutation identification will be described. In an initial study of 12 patients clinically diagnosed with ARVC but not yet genotypically characterised, nine desmosome protein mutations were identified, of which seven were novel.

In addition, we have used next-generation sequencing to discover new genes linked with the regulation of desmosome function. For example, we studied two siblings with autosomal recessive neonatal inflammatory skin and bowel lesions. The affected female died suddenly aged 12 of parvovirus B19 myocarditis, and her brother has mild cardiomyopathy. We have identified the first human loss-of-function mutation in *ADAM17* [encoding a disintegrin and metalloproteinase 17, ADAM17, also called 'tumor necrosis factor (TACE)'-converting enzyme)] as the likely underlying cause of this syndrome (Blaydon, Biancheri et al. 2011). Functional studies revealed defective DSG2 processing. We have also recently identified loss-of-function mutations in the gene for protease inhibitor cystatin A (CSTA) as the underlying genetic cause of exfoliative ichthyosis (Blaydon, Nitoiu et al. 2011).

Electron microscopy of patient skin biopsies revealed that the level of skin peeling occurs between the basal and suprabasal layers in these patients. In addition, in vitro modelling suggests that in the absence of cystatin A protein, there is a cell–cell adhesion defect in human keratinocytes that is particularly prominent when cells are subject to mechanical stress. This is the first evidence of a key role for a protease inhibitor in epidermal adhesion within the lower layers of the human epidermis. Further functional studies describing the effect of ADAM17 and CSTA gene mutations in keratinocytes and desmosome biology will be described.

[The two papers below were in press at the time of Heidelberg Heart II.]

Blaydon DC, Biancheri P, Di WL, Plagnol V et al (2011) Inflammatory skin and bowel disease linked to ADAM17 deletion.N Engl J Med 365:1502–1508

Blaydon DC, Nitoiu D, Eckl KM, Cabral RM et al (2011) Mutations in CSTA, encoding Cystatin A, underlie exfoliative ichthyosis and reveal a role for this protease inhibitor in cell–cell adhesion. Am J Hum Genet 89(4):564–571

**A 34 — The living aortic valve: functional and clinical relevance**

Sir Magdi H. Yacoub

Imperial College London, National Heart & Lung Institute, Harefield Heart Science Centre, Harefield, Middlesex UB9 6JH, UK

m.yacoub@imperial.ac.uk

Until recently, heart valves were thought of as passive structures, which are driven by haemodynamic events, with the sole function of guaranteeing unidirectional flow with minimal or no obstruction. Recent evidence has shown that heart valves perform extremely sophisticated functions which depend on their viability and the specific characteristics of their component parts, at tissue, cellular, and molecular levels. This enables the valves to alter their shape, size and mechanical properties during different phases of the cardiac cycle, and to respond to the continuously changing haemodynamic conditions during health and disease. Such changes have been shown to influence myocardial function, coronary flow, and other organ functions.

Currently, the only valve substitute which guarantees long-term viability of the inserted graft is the pulmonary autograft or the Ross operation. A recent prospective randomised trial comparing the results of the Ross operation to non-viable aortic homograft roots showed significantly better survival and quality of life following the Ross operation, which is thought to be largely due to the viability of the pulmonary autograft. Taken together, these findings support the concept of tissue engineering viable tissue valves.

**A 35 — Heart valves — concepts for tissue engineering**

Serghei Cebotari^1^, Igor Tudorache^1^, Anatol Ciubotaru^2^, Dietmar Boethig^1^, Samir Sarikouch^1^, Adelheid Goerler^1^, Artur Lichtenberg^1^, Eduard Cheptanaru^2^, Sergiu Barnaciuc^2^, Anatol Cazacu^2^, Oxana Maliga^2^, Oleg Repin^2^, Liviu Maniuc^2^, Thomas Breymann^1^, Axel Haverich ^1^

(1) Department of Thoracic and Cardiovascular Surgery, Hannover Medical School, Germany

(2) Cardiac Surgery Center, State Medical and Pharmaceutical University, Chisinau, Moldova

haverich.axel@mh-hannover.de

Degeneration of xenografts or homografts is a major cause for reoperation in young patients after pulmonary valve replacement. Here we present the early results of fresh decellularized pulmonary homografts (DPH) implantation compared to glutaraldehyde-fixed bovine jugular vein (BJV) and cryopreserved homografts (CH). Thirty-eight patients with DPH in pulmonary position were consecutively evaluated during the follow-up (up to 5 years), including medical examination, echocardiography, and MRI. These patients were matched according to age and pathology and compared to BJV (*n* = 38) and CH (*n* = 38) recipients. In contrast to BJV and CH groups, echocardiography revealed no increase of transvalvular gradient, cusp thickening, or aneurysmatic dilatation in DPH patients. Over time, DPH valve annulus diameters converge towards normal z-values. Five-year-freedom from explantation was 100% for DPH; 86 ± 8 % and 88 ± 7 % for BJV and CH conduits respectively. Additionally, MRI investigations in 17 DPH patients with follow-up time >2 years were compared to MRI data of 20 BJV recipients. Both patientís groups (DPH and BJV) were at comparable ages (mean 12.7 ± 6.1 versus 13.0 ± 3.0 years old) and have comparable follow-up time (3.7 ± 1.0 versus 2.7 ± 0.9 years). In DPH patients, the mean transvalvular gradient was significantly (*p* = 0.001) lower (11 mmHg) than in the BJV group (23.2mmHg). Regurgitation fraction was 14 ± 3 % and 4 ± 5 % in the DPH and BJV groups respectively. In three DPH recipients, moderate regurgitation was documented postoperatively, and remained unchanged in follow-up. Conclusions: in contrast to conventional homografts and xenografts, decellularized fresh allograft valves showed improved freedom from explantation, provided low gradients in follow-up and exhibited adaptive growth. This concept may be transferred to myocardial tissue engineering, where most interesting results have been obtained already.

**A 36 — Relevance of understanding valve interstitial and endothelial cell biology to tissue engineering heart valves**

Adrian H. Chester

Imperial College London, National Heart & Lung Institute, Harefield Heart Science Centre, Harefield, Middlesex UB9 6JH, UK

a.chester@imperial.ac.uk

The cellular components of heart valves play a key role in the function and durability of these highly dynamic and complex structures. Heart valve interstitial and endothelial cells both possess unique phenotypic characteristics and functional responses, which allow then to maintain the integrity of valve structure in conditions of a rapidly changing mechanical environment. We, and others, have studied how valve cells respond to growth factors and mechanical cues, to promote cell proliferation and extracellular matrix secretion; optimise the dispensability of valve tissue and retard the development of calcific lesions. These properties rely on communication between the cells and the extracellular matrix, as well as paracrine signalling between endothelial and interstitial cells. These events regulate the secretory and contractile capacity of the interstitial cells.

Understanding the biology of valve cells and the valve extracellular matrix is only the first step in an attempt to tissue engineering a heart valve. Comparative assessment of the responses of candidate cells will provide a rationale for using a particular cell type to tissue engineer a valve. Important characteristics are the development of a valve cell phenotype, stability of that phenotype, secretory properties, immunogenicity, and an ability to respond the hemodynamic conditions experienced by valves to promote a stable and durable structure. In this regard, we have examined the phenotypic and functional characteristics of mesenchymal stem cells isolated from the bone marrow and adipose tissue. Our data suggest that these cell types share some properties with valve interstitial cells, and may also be suitable for differentiation into a valve endothelial cell phenotype. Understanding the properties of valve endothelial and interstitial cells that allow them to maintain a healthy valve is required if the function of these cells is to be recapitulated in tissue engineered valves.

**A 37 — Naturally inspired engineering of heart valves: developmental biology meets regenerative medicine**

Jonathan T. Butcher

Department for Biomedical Engineering, Cornell University, Ithaca, NY, USA

jtb47@cornell.edu

Heart valve disease is a serious and growing clinical problem for which the only therapy is prosthetic valve replacement. These nonliving valves are well-suited for the elderly, but for younger patients are poorly tolerated. Particularly in children, there is a significant need for a living valve replacement that can grow and remodel over time. Our lab has focused on understanding the mechanisms by which these valves are "naturally engineered" during embryonic development, in order to identify paradigms that can accelerate the maturation of tissue-engineered replacements. Two ways we do this is by: 1) using signaling programs grounded in natural developmental valve maturation to coax stem cells naturally towards heart valve phenotypes, and 2) identifying and replicating the evolving developmental hemodynamic signals in tissue-engineered conduits. We employ 3D tissue printing as a means for creating anatomically accurate heterogeneous structures for valve replacement. We are also using this same engineering approach to learn more about the hemodynamic causes of congenital heart defects. We have developed a new method for non-invasive microsurgery of embryonic hearts via focused laser photoablation. We show that this technique is capable of creating models of congenital heart defects without genetic mutation. Engineering approaches offer powerful new ways of thinking about developmental biology that can pay significant dividends towards regenerative medicine strategies.

**A 38 — Endothelial plasticity in cardiac valves**

Joyce Bischoff

Children´s Hospital Boston and Harvard Medical School, Boston, MA, USA

joyce.bischoff@childrens.harvard.edu

The endothelium covering the aortic, pulmonary, mitral, and tricuspid valves looks much like the endothelium throughout the vasculature, in terms of general morphology and expression of many endothelial markers. Closer examination, however, reveals important differences and hints of a unique phenotype that reflects the valvular endothelium´s embryonic history and potentially its ability to maintain integrity and function over a lifespan of dynamic mechanical stress. A well-studied property that sets the cardiac valvular endothelium apart is the ability to transition from an endothelial to a mesenchymal phenotype — an event known as EMT. EMT is a critical step during embryonic valvulogenesis; it can occur in post-natal valves (Paranya et al. 2001; Paruchuri et al. 2006), and has recently been implicated in the adaptive response of mitral valve leaflets exposed to a controlled in vivo setting designed to mimic the leaflet tethering that occurs in ischemic mitral regurgitation (Dal-Bianco et al. 2009). This presentation will discuss what is known about valvular endothelial cells, with a particular focus on EMT in post-natal, adult valves. New data on the plasticity of valvular endothelial cells will be presented (Wylie-Sears et al. 2011). The self-renewal and multi-lineage differentiation potential of valvular ECs suggests that at subset of valvular endothelial cells are progenitor cells, which may serve to replenish valvular cells during normal cellular turnover and in response to injury and disease.

Dal-Bianco JP, Aikawa E, Bischoff J, Guerrero JL, Handschumacher MD, Sullivan S, Johnson B, Titus JS, Iwamoto Y, Wylie-Sears J, Levine RA, Carpentier A (2009) Active adaptation of the tethered mitral valve: Insights into a compensatory mechanism for functional mitral regurgitation. Circulation 120:334–342

Paranya G, Vineberg S, Dvorin E, Kaushal S, Roth SJ, Rabkin E, Schoen FJ, Bischoff J (2001) Aortic valve endothelial cells undergo transforming growth factor-beta-mediated and non-transforming growth factor-beta-mediated transdifferentiation in vitro. Am J Pathol 159:1335–1343

Paruchuri S, Yang JH, Aikawa E, Melero-Martin JM, Khan ZA, Loukogeorgakis S, Schoen FJ, Bischoff J (2006) Human pulmonary valve progenitor cells exhibit endothelial/mesenchymal plasticity in response to vascular endothelial growth factor-a and transforming growth factor-beta2. Circ Res 99:861–869

Wylie-Sears J, Aikawa E, Levine RA, Yang JH, Bischoff J (2011) Mitral valve endothelial cells with osteogenic differentiation potential. Arterioscler Thromb Vasc Biol 31:598–607

THE SPECIAL PATIENT CASE

**A 39 — Dualism in cardiac pathogenesis: illicit use of anabolic–androgenic steroids in a shot-put athlete homozygous for a missense desmoglein-2 (Dsg2) mutation**

Werner W. Franke (Presentation and Discussion Leader)

Helmholtz Group for Cell Biology, German Cancer Research Center DKFZ, Im Neuenheimer Feld 280, D-69120 Heidelberg, Germany

w.franke@dkfz.de

In view of the recently increased recognition of the frequency of ARVC/D patients carrying mutations in constitutive molecular components of the cytoskeleton and the specific junctions (composite junctions, *areae compositae*) of the cardiomyocyte-connecting intercalated disk (see, e.g., van Tintelen, A14, as well as Rickelt and Pieperhoff, special article, in this issue), it is now necessary to consider the very high ratio of young persons who take anabolic–androgenic steroids (AAS) for the promotion of muscle growth and for athletic performance enhancements, including athletes as well as "bodybuilders" and persons in show business.

Although cross-striated skeletal muscle is the desired target tissue of this drug abuse, it was not surprising to learn that cross-striated myocardiac muscle can also increase in size and protein mass (for early animal experiments and reviews, see 'Special Reference List'). This possible coincidence of a genetic predisposition and widespread drug abuse is therefore a special subject of concern, notably in connection with discussions of cases of "sudden death" of active or former AAS abusers. As this drug abuse usually occurs in deeply conspirative secrecy and the persons involved, including family members, coaches, dealers, and sports functionaries, will remain silent, it would need a rare and special combination of circumstances to collect and publish the information needed to present a specific case. Therefore, we thank Mr. Gerd L. Jacobs, born April 4, 1960, in Berlin, for permission to present his case at this meeting.

A slightly condensed summary of his life history is presented in the protocol of his interrogation by the police in the city of Berlin for the County Court Prosecutors on February 2 1998 (an English summary of the protocol can be read on the author's website at http://www.dkfz.de/en/helmholtz-zellbiologie/starting-page-Helmholtz.html).

Mr. Jacobs was an active athlete (track and field) in shot-put and discus throw in the Berlin sports club TSC from 1972 to 1984, and finished his sports career as a sports teacher (in the last years of his career he was among the top-ten of the German Democratic Republic, GDR). His closest club team-mates in shot-put included Ulf Timmermann, Olympic gold medal winner, former world record holder and still European record holder. Mr. Jacobs received the oral drug Oral-Turinabol (OT), a 17-α-alkylated steroid, the most frequently used AAS in GDR sports (Franke and Berendonk 1997). He received the pills directly from the coach and was never informed about possible AAS-induced damages or harmful side-effects. The daily dose varied between two and eight pills, i.e., 10–40 mg. In later years, the dosage was reduced to half. The drug was given in two annual "cycles", each for 4 months.

After 1998 the cardiac health of Mr. Jacobs deteriorated dramatically and rapidly, so that on October 1 2004 his heart had to be replaced by a donor heart. The major statement in the histological diagnosis was "high-grade cardiac muscle hypertrophy with secondary myocardiac damages and myocardial disarrangements".

Independently, and not aware of the AAS drug history, Posch et al. (2008) had found that the explanted heart as well as other tissues of Mr. Jacobs contained two missense mutations in the gene encoding desmoglein-2 (V55M and V919G), i.e., one of the two cadherin-type transmembrane glycoproteins of the desmosomal type (Schäfer et al. 1994, 1996), the importance of which for the development and functions in non-epithelial tissues was first demonstrated by Eshkind et al. (2002). Mr. Jacobs has been reported as homozygous for DSG-V55M, and his father has subsequently been found to be heterozygous for this mutation but also suffering from "dilated cardiomyopathy". Ultrastructural alterations have been reported for the intercalated disk junctions of the explanted heart, including electron microscopy (Posch et al. 2008).

As similar heart problems, including hypertrophic and dilated cardiomyopathies, have also been found in AAS-treated power athletes and bodybuilders (see 'Special Reference List'), the combinations of both situations, i.e. gene mutations in one of the constitutive molecules of the composite junctions (*areae compositae*) of the myocardial intercalated disks (for Dsg2 see, for example, Borrmann et al. 2006; Franke et al. 2006; for further recent reports on Dsg2 mutations in cardiac diseases, see the reference thesaurus in the article by Pieperhoff and Rickelt in this issue) and AAS-treatment (one of the most spectacular cases in Berlin was that of the former shot-put European vice champion Ralf Reichenbach, 48 years old), have to be considered and discussed as a special possible cause for the specific pathogenic developments.

Eshkind L, Tian Q, Schmidt A, Franke WW, Windoffer R, Leube RE (2002) Loss of desmoglein 2 suggests essential functions for early embryonic development and proliferation of embryonal stem cells. Eur J Cell Biol 81:592–598

Franke WW, Berendonk B (1997) Hormonal doping and androgenization of athletes: a secret program of the German Democratic Republic government. Clin Chem 43:1262–1279

Posch MG, Posch MJ, Geier C, Erdmann B, Mueller W, Richter A, Ruppert V, Pankuweit S, Maisch B, Perrot A, Buttgereit J, Dietz R, Haverkamp W, Özcelik C (2008) A missense variant in desmoglein-2 predisposes to dilated cardiomyopathy. Mol Genet Metab 95:74–80

Schäfer S, Koch PJ, Franke WW (1994) Identification of the ubiquitous human desmoglein, Dsg2, and the expression catalogue of the desmoglein subfamily of desmosomal cadherins. Exp Cell Res 211:391–399

Schäfer S, Stumpp S, Franke WW (1996) Immunological identification and characterization of the desmosomal cadherin Dsg2 in coupled and uncoupled epithelial cells and in human tissues. Differentiation 60:99–108

**Special Reference List**

Appell H-J, Heller-Umpfenbach B, Feraudi M, Weicker H (1983) Ultrastructural and morphometric investigations on the effects of training and administration of anabolic steroids on the myocardium of guinea pigs. Int J Sports Med 4:268–274

Behrendt H (1977) Effect of anabolic steroids on rat heart muscle cells. Cell Tissue Res 180:303–315

Behrendt H, Boffin H (1977) Myocardial cell lesions caused by an anabolic hormone. Cell Tissue Res 181:423–426

De Piccoli B, Giada F, Benettin A, Sartori F, Piccolo E (1991) Anabolic steroid use in body builders: an echocardiographic study of left ventricle morphology and function. Int J Sports Med 12:408-412

Di Bello V, Giorgi D, Bianchi M, Bertini A, Caputo MT, Valenti G., Furioso O, Alessandri L, Paterni M., Giusti C (1999) Effects of anabolic-androgenic steroids on weight-lifters' myocardium: an ultrasonic videodensitometric study. Med Sci Sports Exerc 31:514-521

Dickerman RD, Schaller F, Prather I, McConathy WJ (1995) Sudden cardiac death in a 20-year-old bodybuilder using anabolic steroids. Cardiology 86:172–173

Dickerman RD, Schaller F, Zachariah NY, McConathy WJ (1997) Left ventricular size and function in elite bodybuilders using anabolic steroids. Clin J Sport Med 7:90–93

Ferrera PC, Putnam DL, Verdile VP (1997) Anabolic steroid use as the possible precipitant of dilated cardiomyopathy. Cardiology 88:218–220

Fineschi V, Baroldi G, Monciotti F, Paglicci Reattelli L, Turillazzi E (2001) Anabolic steroid abuse and cardiac sudden death: a pathologic study. Arch Pathol Lab Med 125:253–255

Fineschi V, Riezzo I, Centini F, Silingardi E, Licata M, Beduschi G, Karch SB (2007) Sudden cardiac death during anabolic steroid abuse: morphologic and toxicologic findings in two fatal cases of bodybuilders. Int J Legal Med 121:48–53

Furlanello F, Bentivegna S, Cappato R, De Ambroggi L (2003) Arrhythmogenic effects of illicit drugs in athletes (review). Ital Heart J 4:829–837

Hausmann R, Hammer S, Betz P (1998) Performance enhancing drugs (doping agents) and sudden death — a case report and review of the literature. Int J Legal Med 111:161–164

Huie MJ (1994) An acute myocardial infarction occurring in an anabolic steroid user. Med Sci Sports Exerc 26:408-413

Karila TAM, Karjalainen JE, Mäntysaari MJ, Viitasalo MT, Seppälä TA (2003) Anabolic androgenic steroids produce dose-dependent increase in left ventricular mass in power athletes, and this effect is potentiated by concomitant use of growth hormone. Int J Sports Med 24:337–343

Kennedy MC, Lawrence C (1993) Anabolic steroid abuse and cardiac death. Med J 158:346–348

Kindermann W (2006) [Cardiovascular side effects of anabolic-androgenic steroids]. Herz 31:566–573 [Article in German]

Kistler L (2006) Todesfälle bei Anabolikamissbrauch. Todesursache, Befunde und rechtsmedizinische Aspekte. MD (Dr. med.) Thesis, Medical Faculty of Ludwig-Maximilian University, Munich, Germany, 96 pp [Article in German]

Ledl-Kurkowski E, Niebauer (2009) Doping und seine Auswirkungen auf das kardiovaskuläre System. J Kardiol 16:345–150 [Article in German]

Luke JL, Farb A, Virmani R, Sample RH (1990) Sudden cardiac death during exercise in a weight lifter using anabolic androgenic steroids: pathological and toxicological findings (case report). J Forensic Sci 35:1441–1447

Madea B, Grellner W (1996) Langzeitfolgen und Todesfälle bei Anabolikaabusus. Rechtsmedizin 6:33–38 [Article in German]

McKillop G, Todd IC, Ballantyne D (1986) Increased left ventricular mass in a bodybuilder using anabolic steroids. Br J Sports Med 20:151–152

McNutt RA, Ferenchick GS, Kirlin PC, Hamlin NJ (1988) Acute myocardial infarction in a 22-year-old world class weight lifter using anabolic steroids (case report). Am J Cardiol 62:164

Morano I, Weicker H (1985) [Effects of continuous body stress and methandienone on the degradation of myofibrillar proteins in the guinea pig heart and soleus muscle]. Arzneimittelforschung 35:501–503 [Article in German]

Nieminen MS, Ramo MP, Viitasalo M, Heikkila P, Karjalainen J, Mantysaari M, Heikkila J (1996) Serious cardiovascular side effects of large doses of anabolic steroids in weight lifters. Eur Heart J 17:1576–1583

Payne JR, Kotwinski PJ, Montgomery HE (2004) Cardiac effects of anabolic steroids. Heart 90:473–475

Rickelt S, Pieperhoff S (2012) Mutations with pathogenic potential in proteins located in or at the composite junctions of the intercalated disk connecting mammalian cardiomyocytes: a reference thesaurus for arrhythmogenic cardiomyopathies as well as Naxos and Carvajal diseases. Cell Tissue Res, in press in this issue

Rockhold RW (1993) Cardiovascular toxicity of anabolic steroids. Annu Rev Pharmacol Toxicol 33:497–520

Sachtleben TR, Berg KE, Elias BA, Cheatham JP, Felix GL, Hofschire PJ (1993) The effects of anabolic steroids on myocardial structure and cardiovascular fitness. Med Sci Sports Exerc 25:1240–1255

Schollert PV, Bendixen PM (1993) [Dilated cardiomyopathy in a user of anabolic steroids]. Ugeskr Laeger 155:1217–1218 [Article in Danish]

Vogt AM, Geyer H, Jahn L, Schänzer W, Kübler W (2002) [Cardiomyopathy associated with uncontrolled self medication of anabolic steroids]. Z Kardiol 91:357–362 [Article in German]


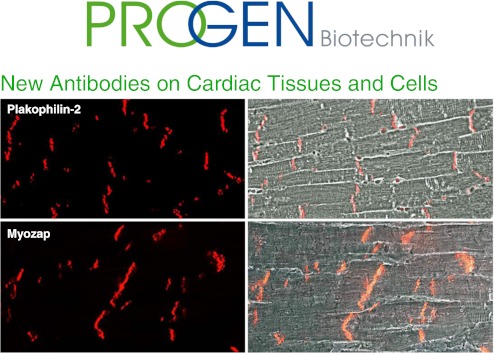


Immunofluorescence microscopy of plakophilin-2 (# 651167) and protein myozap (# 651169) in mammalian heart tissue: Specific decoration of the composite junctions in the intercalated disks.

# 61075 anti-Cardiac a-Actin, mouse mc

# 61001 anti-a-smooth muscle Actin, mouse mc

# 16109 anti-CD34, mouse mc

# 16105 anti-CD117, mouse mc

# 10519 anti-Desmin, mouse mc

# 10570 anti-Desmin, rabbit pc

# 610120 anti-Desmocollin 2, rabbit pc

# 61059 anti-Desmoglein 2, mouse mc

# 651118 anti-Desmoglein 2, mouse mc

# 651119 anti-Desmoglein 2, mouse mc

# 610121 anti-Desmoglein 2, rabbit pc

# DP-1 anti-Desmoplakin 1, guinea pig pc

# 61024 anti-Desmoplakin 1, mouse mc

# 61003 anti-Desmoplakin 1/2, mouse mc

# 65146 anti-Desmoplakin, mouse mcs

# 651155 anti-Desmoplakin 1/2, mouse mc

# 651109 anti-Desmoplakin 1/2, mouse mc

# 651138 anti-Lamin A/C, mouse mc

# 651169 anti-MyoZap, mouse mc

# GP31 anti-MLDP/OXPAT, C-terminus, guinea pig pc

# GP44 anti-MLDP/OXPAT, N-terminus, guinea pig pc

# 61005 anti-Plakoglobin, mouse mc

# 651101 anti-Plakophilin 2, mouse mc

# 651167 anti-Plakophilin 2, mouse mc

# GP-PP2 anti-Plakophilin 2, guinea pig pc

# 61013 anti-Vimentin, mouse mc

# GP53 anti-Vimentin, guinea pig pc

QC certified by C. Kuhn & W.W. Franke (German Cancer Research Center)

**PROGEN** Biotechnik GmbH Maaßstraße 30 69123 Heidelberg Germany

T: +49 6221 8278-0 F: +49 6221 827824 E: **info@progen.de**

H: **www.progen.de**

**Abstracts of poster presentations**

**P 1 — Study of the molecular pathogenesis of arrhythmogenic cardiomyopathy due to Desmoglein-2 mutations: the zebrafish helps the mouse**

Giorgia Beffagna^1^, Alessandra Lorenzon^1^, Martina Milanetto^1,2^, Roberto Doliana^3^, Giuseppe Lembo^4^, Patrizia Sabatelli^5^, Daniela Carnevale^4^, Paolo Grumati^2^, Emanuela Dazzo^1^, Enrico Moro^1^, Francesco Argenton^1^, Paolo Bonaldo^2^, Paola Braghetta^2^, Alessandra Rampazzo^1^

(1) Department of Biology, University of Padova, Padova, Italy

(2) Department of Histology, Microbiology, and Medical Biotechnology, University of Padova, Padova, Italy

(3) Department of Molecular Oncology and Translational Research, CRO-IRCCS, Aviano, Pordenone, Italy

(4) Department of Angio-Cardio-Neurology, Neuromed Institute Pozzilli, Italy

(5) Unit of Bologna c/o IOR, Division of Experimental Oncology, Bologna, Italy

alessandra.rampazzo@unipd.it

Arrhythmogenic right ventricular cardiomyopathy (ARVC) is a progressively degenerative cardiomyopathy, frequently involved in juvenile sudden death. ARVC is considered a disease of cell adhesion, because mutations in desmosomal genes have been involved in the pathogenesis of ARVC in a significant proportion of patients. To identify the molecular pathogenic mechanisms involved in ARVC, we have generated cardiac-restricted desmoglein-2 (DSG2) transgenic mice. All the transgenic lines expressing human mutated DSG2 (G100R, N266S, Q558X) resemble the clinical features of ARVC. At the molecular level, we checked the expression and localization of some adhesion proteins, demonstrating changes in expression and localization of beta-catenin in hearts of transgenic mice carrying the mutated proteins. To establish whether the observed increase in beta-catenin expression has a possible direct effect on Wnt signaling, we co-injected in zebrafish embryos the WT and mutated DSG2 mRNAs with BAT-lux reporter plasmids containing the luciferase enzyme cDNA under the control of the responsive elements of Wnt/beta-catenin signaling. Luciferase reporter activity occurred significantly higher in fishes injected with mutant constructs than in those injected with wild type.

To further examine the relationship of beta-catenin and Wnt signaling, we co-injected dickkopf-1 (Dkk1), an inhibitor of Wnt signalling, and we showed that Dkk1 rescued the phenotype in embryos. In conclusion, mutations in desmosomal proteins can perturb the normal balance of critical proteins in junctions and the citosol which, in turn, could alter gene expression by circumventing normal Wnt signaling pathway.

**P 2 — Conduction slowing and arrhythmogenesis in desmoglein-2 mutant mice due to intercalated disk abnormalities, prior to the development of cardiomyopathic changes**

Stefania Rizzo^1^, Elisabeth M Lodder^2^, Carol Ann Remme^2^, Rianne Wolswinkel^2^, Kalliopi Pilichou^1^, Cristina Basso^1^, Gaetano Thiene^1^, Connie R Bezzina^2^

(1) University of Padua, Padua, Italy

(2) University of Amsterdam, Amsterdam, The Netherlands

stefania.rizzo@studenti.unipd.it; e.m.lodder@amc.uva.nl

We investigated whether intercalated disk (ID) remodelling in arrhythmogenic cardiomyopathy due to desmosomal mutations impacts on cardiac elecrophysiological properties before the onset of structural changes. We studied transgenic mice with low cardiac overexpression of N271S-dsg2 (TgNS/L, the mouse homolog of the human mutation DSG2-N266S) at three different ages: <2 wks, 3–4 wks and >6wks. Mice with cardiac overexpression of wild-type dsg2 (TgWT) and wild-type served as controls. ECG and epicardial mapping were performed to determine ventricular conduction and arrhythmia susceptibility. The structure and molecular composition of the ID was assessed by electron microscopy (EM) and by immunofluorescence. Cardiomyopathic changes were observed by EM in Tg-NS/L only from >6 wks. At ECG, QRS-prolongation and spontaneous arrhythmias were observed in TgNS/L only from age >6 wks. However, on epicardial mapping, ventricular activation time was prolonged in TgNS/L at 3–4 wks (mean ± SEM 11.9 ± 1.8 ms, *n* = 5) compared to WT (7.6 ± 0.6, *n* = 4) and TgWT (7.4 ± 0.5, *n* = 5) (*p* < 0.05). In addition in this same age group, ventricular arrhythmias were inducible in TgNS/L, but not in controls. EM uncovered gap-widening at desmosomes /adherens junctions, but not in the gap junctions, starting at 3–4 wks exclusively in TgNS/L mice. No differences in the level and localization of junctional proteins were found between TgNS/L and controls. Dsg-2 mutant mice display conduction slowing and ventricular arrhythmias before development of cardiomyopathic changes. This coincided with the time-point at which junctional gap-widening was observed, suggesting that ID integrity is required for proper electrical conduction.

**P 3 — Development and evaluation of a perfusion decellularization porcine heart model: generation of three-dimensional myocardial neoscaffolds**

Alexander Weymann^1^, Bastian Schmack^1^, Carsten Schies^1^, Nicole Chaimow^1^, Ines Pätzold^1^, Benjamin Claus^2^, Kristóf Hirschberg^1,3^, Pál Soós^1,3^, Sivakkanan Loganathan^1^, Enikö Barnucz^1^, Sevil Korkmaz,^1^ Pascal Dohmen^4^, Gábor Szabó^1^, Matthias Karck^1^

(1) Department of Cardiac Surgery, Heart Center, University of Heidelberg, Heidelberg, Germany

(2) Department of Cardiovascular Surgery, Charité–University Medicine Berlin, Campus Charité Mitte, Berlin, Germany

(3) Heart Center Semmelweis University, Semmelweis University, Budapest, Hungary

(4) Department of Cardiac Surgery, Heart Center, University of Leipzig, Leipzig, Germany

Alexander.Weymann@med.uni-heidelberg.de; Matthias.Karck@med.uni-heidelberg.de

Reports about the generation of three-dimensional neoscaffolds for myocardial tissue engineering are limited. The architecture provided by perfusion decellularization of whole human-sized hearts would support the production of three-dimensional living tissues from an acellular matrix. The aim of this study was to evaluate the potential of a perfusion decellularization model for whole-heart tissue engineering.

Hearts were obtained from 12 German Landrace pigs from a selected abattoir. After preparation, the hearts were mounted and perfused on a modified Langendorff decellularization model specifically constructed for this reason. Decellularization was achieved by an ionic detergent-based perfusion protocol. The quality of the decellularization process was quantified by histology and fluorescence microscopy. Data concerning the presence of residual DNA within the decellularized hearts were measured with spectrophotometric quantification and compared to controls. For the measurement of mechanical stability, a micromanometer was advanced into the ventricle and determined LV pressure at different LV volumes.

The here-described protocol preserved the micro- and macroarchitecture of the hearts. After histological examination, all hearts lacked intracellular components but retained various types of collagen, proteoglycan, and elastin. Quantitative DNA analysis demonstrated a significant reduction of DNA in decellularized hearts compared to controls (84.32 ± 3.99ng DNA/mg tissue vs 470.13 ± 18.77ng DNA/mg tissue (*p* < 0.05)). Using the decellularization protocol described in the present study, decellularized hearts showed similar mechanical stability as native hearts. The modified Langendorff perfusion decellularization model described here is applicable for whole porcine hearts by removing cellular content and DNA. The resulting three-dimensional matrix provides an interesting tool for further studies in the field of whole heart tissue engineering.

**P 4 — Tissue engineering of the stented pericardium using magnetic guidance for recellularization**

Ali Ghodsizad^1^, Viktor Bordel^1^, Jose Berjon Gonzalez^2^, Matthias Karck^1^, Arjang Ruhparwar^1^

(1) Department of Cardiac Surgery, University of Heidelberg, Germany

(2) Department of Pathology, The Methodist Hospital, Houston, TX, USA

Matthias.Karck@med.uni-heidelberg.de

Application of progenitor cells for differentiation in a three-dimensional pattern is an important aspect in tissue engineering. We report on development of a bioreactor for three-dimensional cell culture experiments using magnetically guided cell recellularization under simulated physiologic conditions.

Stented pericardial aortic valves were constructed and decellularized by detergents. Isolated, magnetically labelled human cord-blood-derived unrestricted somatic stem cells (USSCs) were cultured in the bioreactor for up to 130 hours (*n* = 8). A magnetic field was created around the valve within the bioreactor for specific delivery of circulating cells to their desired targets. In the control group, the magnetic field was absent. The bioreactor enabled the retention of physiologic culture conditions with high pulsatile flows , aerobic cell metabolism oxygen tension (pO2 — 130 ± 20 mmHg) and physiological pH values (7.4 ± 0.05) during the whole experimental procedure. The histological characterization of the matrix and cells was assessed by immunohistochemical staining and electronic microscopy. Macroscopic and histological analysis showed a homogeneous seeding of the valve with USSCs in the magnet group, but no seeding of cells could be observed in the non-magnetic group.

Our modified multifunctional bioreactor allows for three-dimensional culturing of human cord-blood-derived unrestricted somatic stem cells on pericardial stented valves with the support of magnetic cell guidance.

**P 5 — Osteopontin and CD44 synthesis are increased in fibrotic hearts of desmoglein 2 mutant mice**

Phillip Krull, Sebastian Kant, Rudolf E. Leube, Claudia A. Krusche

Institute of Molecular and Cellular Anatomy, Medical Faculty, RWTH Aachen University, Aachen, Germany

ckrusche@ukaachen.de; rleube@ukaachen.de

Desmoglein 2 (DSG2) mutant mice develop dilative cardiomyopathy characterized by cardiomyocyte necrosis, calcification, and fibrosis (Krusche, Holthöfer et al., 2011). Since osteopontin (OPN) is involved in calcification, inflammation, and cardiac fibrosis (Renault et al. 2010; Matsui et al. 2004; Okamoto et al. 2011), we studied expression and tissue distribution of OPN and its receptor, the CD44 antigen, which is involved in leukocyte recruitment (Weber et al. 1996; DeGrendele et al. 1996). mRNA synthesis was assessed by real-time RT-PCR, and proteins were localized by immunohistochemistry. Wild-type and homozygous DSG2 mutant mice were compared. At 2 weeks, OPN and CD44 mRNA synthesis was significantly elevated in mutants already displaying cardiac lesions. Mutants without pathology showed wild-type expression levels. At 8 and 12 weeks, all mutant hearts showed fibrotic alterations. The OPN and CD44 mRNA synthesis was higher in the mutant than in the wildtype. OPN protein was localized exclusively in necrotic cardiomyocytes. CD44+ cells were primarily localized in fibrotic lesions with necrotic cardiomyocytes. The highest CD44+ cell density was found in lesions of 2- and 4-week-old mutants. Later, CD44+ cell density declined, but did not reach wild-type levels. Staining of serial sections with the immune cell marker CD45 implies that a high proportion of the CD44+ cells are immune cells. In conclusion, in DSG2 mutant mice cardiac OPN expression is associated with the onset of cardiac necrosis and fibrosis and the perpetuation of this process. This is in accordance with observations reported for desmin -/- mice (Mavroidis et al., 2002). In addition, CD44+/CD45+ cells are involved in the acute and chronic phases of the cardiomyopathy.

DeGrendele HC, Estess P, Picker LJ, Siegelman MH (1996) CD44 and its ligand hyaluronate mediate rolling under physiologic flow: a novel lymphocyte-endothelial cell primary adhesion pathway. Exp Med 183:1119–1130

Krusche CA, Holthöfer B, Hofe V, van de Sandt AM, Eshkind L, Bockamp E, Merx MW, Kant S, Windoffer R, Leube RE (2011) Desmoglein 2 mutant mice develop cardiac fibrosis and dilation. Basic Res Cardiol 106:617–633

Matsui Y, Jia N, Okamoto H, Kon S, Onozuka H, Akino M, Liu L, Morimoto J, Rittling SR, Denhardt D, Kitabatake A, Uede T (2004) Role of osteopontin in cardiac fibrosis and remodeling in angiotensin II-induced cardiac hypertrophy. Hypertension 43:1195–1201

Mavroidis M, Capetanaki Y (2002) Extensive induction of important mediators of fibrosis and dystrophic calcification in desmin-deficient cardiomyopathy. Am J Pathol 160:943–952

Okamoto H, Imanaka-Yoshida K (2011) Matricellular proteins: new molecular targets to prevent heart failure. Cardiovasc Ther [Epub ahead of print]

Renault MA, Robbesyn F, Réant P, Douin V, Daret D, Allières C, Belloc I, Couffinhal T, Arnal JF, Klingel K, Desgranges C, Dos Santos P, Charpentier F, Gadeau AP (2010) Osteopontin expression in cardiomyocytes induces dilated cardiomyopathy. Circ Heart Fail 3:431–439

Weber GF, Ashkar S, Glimcher MJ, Cantor H (1996) Receptor–ligand interaction between CD44 and osteopontin (Eta-1). Science 271:509–512

**P 6 — Desmoglein 2-mutant mice develop dilated cardiomyopathy without changes in the protein synthesis of the arm-repeat domain proteins plakoglobin, plakophilin 2, and β-catenin**

Sebastian Kant, Claudia A. Krusche, Rudolf E. Leube

Institute of Molecular and Cellular Anatomy, Medical Faculty, RWTH Aachen University, Aachen, Germany

skant@ukaachen.de; ckrusche@ukaachen.de; rleube@ukaachen.de

Desmosomal proteins are important intercalated disc (ID) components coupling cardiomyocytes. In humans, mutations of desmosomal proteins have been implicated in arrhythmogenic right ventricular cardiomyopathy (ARVC) and also in dilated cardiomyopathy (DCM), which are both characterized by fibrosis and arrhythmia (Awad et al. 2008; Posch et al. 2008; Basso et al. 2008; Marcus et al. 2010). To investigate the pathomechanism of desmosome-related cardiomyopathy, we developed mice carrying a deletion in the extracellular domains of the desmosomal cadherin desmoglein 2 (Dsg2). As expected, these mice develop cardiomyopathy with dilation, pronounced fibrosis and arrhythmia. It has been suggested that alterations in the ID localization of the plaque protein plakoglobin are reliable diagnostic criteria for ARVC, with important implications for its pathogenesis occurring in concert with the related *arm*-repeat proteins plakophilin 2 and β-catenin (Garcia-Gras et al. 2006; Asimaki et al. 2009; Fabritz et al. 2011). We therefore examined the protein level and distribution of these polypeptides in our mouse model. For comparison, we studied the synthesis of desmoplakin and N-cadherin. All of these molecules are localized to the ID in the wildtype. Protein synthesis of wild-type and mutated mouse hearts was quantified by immunoblotting, revealing a reduction of the mutant Dsg2 but no changes of the other polypeptides. To examine the distribution of the respective polypeptides in mutant heart tissue, semiquantitative immunohistology was performed at different stages of disease development and in different areas in relation to the fibrotic lesions. Except for Dsg2, all proteins still localized to the ID at the same stoichiometry as in the wildtype. We conclude that down-regulation or mislocalization of plakoglobin and other *arm* ID proteins is not obligatory for desmosome-dependent cardiomyopathy.

Asimaki A, Tandri H, Huang H, Halushka MK, Gautam S, Basso C, Thiene G, Tsatsopoulou A, Protonotarios N, McKenna WJ,Calkins H, Saffitz JE (2009) A new diagnostic test for arrhythmogenic right ventricular cardiomyopathy. N Engl J Med 360:1075–1084

Awad MM, Calkins H, Judge DP (2008) Mechanisms of disease: molecular genetics of arrhythmogenic right ventricular dysplasia/cardiomyopathy. Nat Clin Pract Cardiovasc Med. 5:258–267

Basso C, Ronco F, Marcus F, Abudureheman A, Rizzo S, Frigo AC, Bauce B, Maddalena F, Nava A, Corrado D, Grigoletto F, Thiene G (2008) Quantitative assessment of endomyocardial biopsy in arrhythmogenic right ventricular cardiomyopathy/dysplasia: an in vitro validation of diagnostic criteria. Eur Heart J 29:2760–2771

Fabritz L, Hoogendijk MG, Scicluna BP, van Amersfoorth SC, Fortmueller L, Wolf S, Laakmann S, Kreienkamp N, Piccini I,Breithardt G, Noppinger PR, Witt H, Ebnet K, Wichter T, Levkau B, Franke WW, Pieperhoff S, de Bakker JM, Coronel R, Kirchhof P (2011) Load-reducing therapy prevents development of arrhythmogenic right ventricular cardiomyopathy in plakoglobin-deficient mice. J Am Coll Cardiol 57:740–750

Garcia-Gras E, Lombardi R, Giocondo MJ, Willerson JT, Schneider MD, Khoury DS, Marian AJ (2006) Suppression of canonical Wnt/beta-catenin signaling by nuclear plakoglobin recapitulates phenotype of arrhythmogenic right ventricular cardiomyopathy. J Clin Invest 116:2012–2021

Marcus FI, McKenna WJ, Sherrill D, Basso C, Bauce B, Bluemke DA, Calkins H, Corrado D, Cox MG, Daubert JP, Fontaine G, Gear K, Hauer R, Nava A, Picard MH, Protonotarios N, Saffitz JE, Sanborn DM, Steinberg JS, Tandri H, Thiene G, Towbin JA, Tsatsopoulou A, Wichter T, Zareba W (2010) Diagnosis of arrhythmogenic right ventricular cardiomyopathy/dysplasia: proposed modification of the Task Force Criteria. Eur Heart J 31:806–814

Posch MG, Posch MJ, Geier C, Erdmann B, Mueller W, Richter A, Ruppert V, Pankuweit S, Maisch B, Perrot A, Buttgereit J, Dietz R, Haverkamp W, Özcelik C (2008) A missense variant in desmoglein-2 predisposes to dilated cardiomyopathy. Mol Genet Metab 95:74–80

**P 7 — Ultrastructural changes in hearts of desmoglein 2-mutant mice**

Sabine Eisner, Claudia A. Krusche, Sebastian Kant and Rudolf E. Leube

Institute of Molecular and Cellular Anatomy, Medical Faculty, RWTH Aachen University, Aachen, Germany

ckrusche@ukaachen.de; rleube@ukaachen.de

Mice carrying a deletion of the extracellular EC1/EC2-domains of the desmosomal cadherin desmoglein 2 develop dilated cardiomyopathy with arrhythmia and fibrosis (Krusche, Holthöfer et al. 2011). The mutant desmoglein 2 is still targeted to the intercalated disc region together with the other desmosomal proteins, albeit at reduced amounts (Krusche, Holthöfer et al. 2011). This suggests that altered adhesion may be responsible for initiation of the disease process upon continued mechanical stress. To examine this idea, cardiomyocyte ultrastructure was examined. We find that, in contrast to the wild type, prominent desmosomes are rather scarce. Widening of the intercellular cleft occurs with complete dissociation at later time points. In addition, sarcomeric structure is disturbed, starting with altered Z-discs. At later disease stages, mitochondria are swollen and multiple lamellar bodies are found in the cytoplasm. Finally, calcifying necrosis of cardiomyocytes and substitution by connective tissue is observed. Taken together, the electron microscopic data support the notion that the mutant desmoglein 2 leads to reduced adhesion in the intercalated disc region, which then leads to secondary changes in sarcomeric structure and cell viability, resulting in necrotic cell death and fibrosis.

Krusche CA, Holthöfer B, Hofe V, van de Sandt AM, Eshkind L, Bockamp E, Merx MW, Kant S, Windoffer R, Leube RE (2011) Desmoglein 2 mutant mice develop cardiac fibrosis and dilation. Basic Res Cardiol 106:617–633

**P 8 — The adhering junctions of mammalian valvular interstitial cells: characterization of fetal and adult heart valves in situ and in culture**

Mareike Barth^1*^, Payam Akhyari^2^, Artur Lichtenberg^2^, Werner W. Franke^1^

(1) Helmholtz Group for Cell Biology, German Cancer Research Center, Im Neuenheimer Feld 280, D-69120 Heidelberg, Germany

(2) Department of Cardiovascular Surgery, Düsseldorf University Hospital, Moorenstrasse 5, D-40225 Düsseldorf, Germany

^*^present address: Institute for Pharmacology and Clinical Pharmacology, Düsseldorf University Hospital, Universitätsstraße 1, D-40225 Düsseldorf, Germany

Mareike.Barth@uni-duesseldorf.de; w.franke@dkfz.de

Tissue-engineered heart valves are in high demand in cardiovascular surgery. Despite remarkable recent progress in the design and preparation of artificial valve scaffolds or of scaffolds seeded with certain kinds of cells in vitro, cell and molecular biological knowledge about valvular interstitial cells (VICs), i.e., the cells originally populating the heart valve, is astonishingly limited, in particular with respect to their special cell–cell junctions.

Using cell biological, biochemical, and immunofluorescence microscopy methods as well as (immuno-)electron microscopy, we have characterized the adhering junctions of VICs of human, bovine, and ovine origin in situ and after proliferation in cell culture. These junctions are of the adherens junction (AJ) type, as demonstrable by electron microscopy, and are similar to those found in other mesenchymal tissues. They are composed of the transmembrane glycoproteins N-cadherin and cadherin-11 and the cytoplasmic plaque proteins α- and β- catenin, plakoglobin and p120ctn. Desmosomal proteins are absent from adult VICs in situ. Surprisingly, however, VICs in 2-D culture rather rapidly acquire an additional plaque protein, the desmosomal protein plakophilin-2 (Barth et al. 2009) which, however, recently has also been noted to occur as a novel acquisition in the AJ plaques of diverse malignantly transformed mesenchymal cells or other cells with increased proliferation rates (Rickelt et al., 2009), cardiax myxomata included (Rickelt et al. 2010). This quite alarming AJ alteration, however, has been shown to be reversible in situ when these VICs are grown in a 3D-environment composed of artificial or decellularized heart valve scaffolds, mimicking the interior of native heart valves, as well as in surgically implanted valve replacement structures. In pathologically altered heart valve tissues such as papillary fibroelastoma or myxomatous degenerated heart valves, plakophilin-2 has generally been found to be absent from the AJs present (Barth 2011).

Fetal heart valves of not only human, but also of porcine and ovine origin surprisingly show — besides the ensemble of AJ proteins known to occur in VICs of adult valves — also the very frequent, near-regular occurrence of the additional plaque protein plakophilin-2 in their AJs, both in situ as well as in 2-D cultures. Remarkably, here not only VICs show the additional plakophilin-2 but also endothelial cells of the endocardium covering the heart valve leaflet. Therefore, a possible role for plakophilin-2 as a marker for proliferatively active mesenchymal cells present in fetal VICs as well as in 2-D cultures of adult VICs is discussed.

Barth M (2011) The cell and molecular biological characterization of cell-cell junctions in mammalian heart valves. PhD Thesis. Faculty of Biosciences, University of Heidelberg, Germany. 161 pp

Barth M, Schumacher H, Kuhn C, Akhyari P, Lichtenberg A, Franke WW (2009) Cordial connections: the molecular ensemble and the structures of the adhering junctions connecting interstitial cells of cardiac valves in situ and in cell culture. Cell Tissue Res 337:63–77

Rickelt S, Winter-Simanowski S, Noffz E, Kuhn C, Franke WW (2009) Upregulation of plakophilin-2 and its acquisition to adherens junctions identifies a novel molecular ensemble of cell–cell-attachment characteristic for transformed mesenchymal cells. Int J Cancer 125:2036–2048

Rickelt S, Rizzo S, Dörflinger Y, Zentgraf H, Basso C, Gerosa G, Thiene G, Moll R, Franke WW (2010) A novel kind of tumor type-characteristic junction: plakophilin-2 as a major protein of adherens junctions in cardiac myxomata. Mod Pathol 23:1429–1437

**P 9 — The protein myozap, a major constitutive plaque component of the composite junctions (areae compositae) of the myocardiac intercalated disks, is also a major and widespread protein in the cell–cell junctions of endothelial cells of blood and lymph vessels**

Sebastian Pieperhoff^1,2,^*, Steffen Rickelt^1,3^, Caecilia Kuhn^1,3^, Ralf Zimbelmann^1^, Hans W Heid^1^, Heiderose Schumacher^1^, Stefanie Winter-Simanowski^1^, Norbert Frey^4^, Werner W. Franke^1,3^

(1) Helmholtz Group Cell Biology, German Cancer Research Center (DKFZ), Heidelberg, Germany

(2) Department of Zoology and Faculty of Land and Food Systems, University of British Columbia, Vancouver, BC, Canada

(3) Progen Biotechnik GmbH, Heidelberg, Germany

(4) Department of Cardiology and Angiology, University Hospital Schleswig-Holstein, Campus Kiel, Germany

^*^present address: Centre for Cardiovascular Science, The Queen’s Medical Research Institute, University of Edinburgh, Scotland, UK

s.pieperhoff@gmail.com; w.franke@dkfz.de

The protein myozap, a 54-kDa polypeptide which is not a member of any of the known junctional protein multigene families, has been identified as a major constituent of the plaques of the composite junctions in the intercalated disks (IDs) connecting cardiomyocytes (Seeger et al. 2010). Using highly sensitive and specific antibodies, we have detected myozap also in cytoplasmic plaques of the adherens junctions (AJs) connecting endothelial cells of the mammalian blood and lymphatic vascular system, including the desmoplakin-containing complexus adhaerentes of the virgultar cells of lymph node sinus, and in cultured endothelial cells. In light- and electronmicroscopic immunolocalization experiments, we show that myozap colocalizes with several proteins of desmosomal plaques as well as with AJ-specific molecules, including VE- and N-cadherin. In biochemical analyses of myozap, immunoprecipitation experiments have revealed desmoplakin, plakophilin-2, protein ZO-1 and plectin as the most prominent complex partners. We conclude that myozap is a general component of cellular junctions in the cardiovascular system, suggesting that this protein not only serves a specific role in the heart IDs, but also functions in the vascular system (for details see Pieperhoff et al. 2011).

Seeger TS, Franke D, Rohr C, Will R, Grund C, Koegel M, Franke WW, Katus HA, Olson EN, Frey N (2010) Myozap, a novel intercalated disc protein, activates serum response factor-dependent signaling and is required to maintain cardiac function in vivo. Circ Res 106:880–890

Pieperhoff S, Heid H, Claycomb WC, Zimbelmann R, Kuhn C, Winter-Simanowski S, Kuhn C, Frey N, Franke WW (2011) The plaque protein myozap defines novel major categories of cardiovascular adhering junctions. J Cell Mol Med 13 Oct [Epub ahead of print]

**P 10 — Cell–cell junctions out of the textbook scheme: special structures in their own right**

Steffen Rickelt^1,2^, Sebastian Pieperhoff^1,3^, Caecilia Kuhn^2^, Roland Moll^4^, Werner W. Franke^1,2^

(1) Helmholtz Group for Cell Biology, German Cancer Research Center, Heidelberg, Germany

(2) Progen Biotechnik, Heidelberg

(3) Centre for Cardiovascular Science, The Queen´s Medical Research Institute, University of Edinburgh, Scotland, UK

(4) Institute of Pathology, Philipps University Marburg, Germany

s.rickelt@dkfz.de; w.franke@dkfz.de

According to the prevailing textbook dogma there are two — and only two — distinct types of cell–cell connecting, Ca^2+^-dependent, plaque-bearing adhering junctions (AJs): the microfilament-associated AJs, including the zonula adhaerens and puncta adhaerentia, and the intermediate filament (IF)-anchoring desmosomes (maculae adhaerentes), most prominent for epithelial cells. The molecular composition of both kinds of AJs has been — almost completely — elucidated, and the specific antibodies are widely used in diagnostic pathology and in developmental biology. Using immunocytochemical, electron, and immunoelectron microscopical methods, we have revealed that in addition to these "textbook categories" of junctions a broad range of other junctions exists. These are the tiny puncta adhaerentia minima, the taproot junctions (manubria adhaerentia), the plakophilin-2-containing AJs of mesenchymal or mesenchymally derived cell types, including malignantly transformed ones, as well as the composite junctions (areae compositae) of the mature mammalian myocardium, the cortex adhaerens of the eye lens, the interdesmosomal "sandwich" or "stud" junctions in the subapical layers of stratified epithelia (including tumors derived therefrom), and the complexus adhaerentes of the endothelial and virgultar cells of the vascular system. These junctions cannot be subsumed under one of the major categories mentioned above but represent special structures in their own right, appear to serve special functions, and can may provide new markers for cell typing.

Franke WW, Borrmann CM, Grund C, Pieperhoff S (2006) The area composita of adhering junctions connecting heart muscle cells of vertebrates. I. Molecular definition in intercalated disks of cardiomyocytes by immunoelectron microscopy of desmosomal proteins. Eur J Cell Biol 85:69–82

Franke WW, Rickelt S, Barth M, Pieperhoff S (2009) The junctions that don't fit the scheme: special symmetrical cell–cell junctions of their own kind. Cell Tissue Res 338:1–17

Moll R, Sievers E, Hämmerling, Schmidt A, Barth M, Kuhn C, Grund C, Hofmann I, Franke WW (2009) Endothelial and virgultar cell formations in the mammalian lymph node sinus: endothelial differentiation morphotypes characterized by a special kind of junction (complexus adhaerens). Cell Tissue Res 335:109–141

Pieperhoff S, Barth M, Rickelt S, Franke WW (2010) Desmosomal molecules in and out of adhering junctions: normal and diseased states of epidermal, cardiac and mesenchymally derived cells. Dermatol Res Pract 2010: 139167

Rickelt S, Rizzo S, Dörflinger Y, Zentgraf H, Basso C, Gerosa G, Thiene G, Moll R, Franke WW (2010) A novel kind of tumor type-characteristic junction: plakophilin-2 as a major protein of adherens junctions in cardiac myxomata. Mod Pathol 23:1429–1437

**P 11 — Telocyte connections in the heart**

Mihaela Gherghiceanu, Laurentiu M. Popescu

Department of Advanced Studies, ‘Victor Babeş’ National Institute of Pathology, Bucharest, Romania

mgherghiceanu@yahoo.com

The heart is a heterogeneous organ composed of different types of cardiomyocytes, capillaries, nerves, and a large variety of interstitial cells. Among interstitial cells, telocytes (TCs), cells with telopodes (Tp), seem to be particularly involved in intercellular communication (Popescu and Faussone-Pellegrini 2010). Electron microscopy (EM) studies have shown that TCs are interconnected, and have close apposition but not conventional junctions (Franke et al. 2009) with cardiomyocytes (CM) and other interstitial cells. In order to detail the architecture of the heterocellular and homocellular junctions of TCs in mouse heart we performed electron tomography (ET). EM and ET showed that Tp are coupled each other by different types of homocellular junctions: *puncta adhaerentia minima, processus adhaerentes* and *manubria adhaerentia*. In addition, TCs establish heterocellular junctions with adult cardiomyocytes (Gherghiceanu and Popescu 2011), cardiomyocyte progenitors or putative stem cells (Gherghiceanu and Popescu 2010). EM study also showed that TCs establish small atypical junctions, *point contacts*, with macrophages or with Schwann cells. Ultrastructural analysis showed that TCs form an interstitial network by homocellular junctions, and link all cardiac cells. We assume that TCs play a significant role in cardiac physiology as structural support for long-distance heterocellular signaling.

Franke WW, Rickelt S, Barth M, Pieperhoff S (2009) The junctions that don't fit the scheme: special symmetrical cell–cell junctions of their own kind. Cell Tissue Res 338:1–17

Gherghiceanu M, Popescu LM (2010) Cardiomyocyte precursors and telocytes in epicardial stem cell niche: electron microscope images. J Cell Mol Med 14:871–877

Gherghiceanu M, Popescu LM (2011) Heterocellular communication in the heart: electron tomography of telocyte–myocyte junctions. J Cell Mol Med 15:1005–1011

Popescu LM, Faussone-Pellegrini MS (2010) Telocytes — a case of serendipity: the winding way from interstitial cells of Cajal (ICC), via interstitial Cajal-like cells (ICLC) to telocytes. J Cell Mol Med 14:729–740

**P 12 — Intermediate filaments are essential for the structure and function of the cardiac conduction system (sinus node)**

Nikos Athanasiadis, Dimitris Chaniotis, Pavlos Rigas, Ioanna Kostavsili, Erene Skaliora, Yassemi Capetanaki, Costas Davos, Manolis Mavroidis

Biomedical Research Foundation, Academy of Athens, Greece

emavroeid@bioacademy.gr

In mammals, each heartbeat is triggered by a single electrical impulse generated in the "pacemaker region" of the sinus node, and then propagated to the atrial and ventricular myocytes. In the present study, we have tried to elucidate how perturbations of the intermediate filament (IF) cytoskeleton lead to cardiac conduction system dysfunction and arrhythmias. As is known, the cardiac phenotype of mutations in the muscle specific IF protein desmin can involve — besides others — conduction defects such as AV block and ARVD/C. In wild-type sinus nodes, we have found that transitional cells are laterally coupled by structures we call "lateral intercalated discs". By immunofluorescence staining, these structures are positive for desmoplakin, ‚-catenin and desmin. These structures are also evident by electron microscopy. Surprisingly, analysis of sinus node isolated from desmin-null mice has indicated that "lateral intercalated discs" are diminished or absent in these mice. In addition, analysis of electrical activity (field potential recordings) of isolated sinus nodes has revealed that there is a 50 % reduction in spike amplitude and a 40 % increase in discharge frequency in desmin-null mice compared to the wild-type one. These results support the hypothesis that cells in the desmin-null sinus node have decreased communication and coordination capacity compared to wild type.

Analysis of sinus nodes from human healthy individuals who unfortunately died young revealed that these "lateral intercalated disks" are also present in transitional cells, and we have found (by immunofluorescence) that desmoplakin is a major constituent of the subsarcolemmal cytoskeleton. Desmoplakin forms a lattice at the subsarcolemmal level (costameres), possibly providing a scaffold with very important functions for the human conduction system. Sinus nodes from young individuals who died from sudden cardiac death are under analysis as candidates with plausible perturbations in these elements.

**P 13 — Deleterious effects of the** ***TMEM43*** **mutation p.S358L found in a German family with arrhythmogenic right ventricular cardiomyopathy and sudden cardiac death**

Baerbel Klauke^1^, Carolin Baecker^1^, Joerg Muesebeck^2^, Eric Schulze-Bahr^3^, Désirée Gerdes^1^,

Anna Gaertner^1^, Hendrik Milting^1^

(1) Heart and Diabetes Center NRW, Erich & Hanna Klessmann-Institute for Cardiovascular Research & Development, Bad Oeynhausen, Germany

(2) University of Bremen, Center of Human Genetics, Bremen, Germany

(3) University Clinic Münster, Institute for Genetics of Heart Diseases, Münster, Germany

bklauke@hdz-nrw.de; hmilting@hdz-nrw.de

Arrhythmogenic right ventricular cardiomyopathy (ARVC) is characterized by loss of ventricular myocardium, which might lead to terminal heart failure or sudden cardiac death (SCD). ARVC is a genetically heterogeneous disorder. The purpose of this study was to determine the prevalence of ARVC related *TMEM43* gene mutations in 22 ARVC patients.

We screened the entire *TMEM43* gene of 22 ARVC index patients previously genotyped for desmosomal (*DSG2, DSC2, PKP2, JUP, DSP*) and *DES* gene mutations (Klauke et al. 2010) by denaturing high pressure liquid chromatography (dHPLC) and Sanger sequencing. We have identified a 70-year-old female with borderline ARVC (Sen-Chowdhry et al. 2010) as carrier of the previously identified *TMEM43* c. 1073C>T (p.S358L) mutation (Merner et al. 2008; Christensen et al. 2011). Cosegregation with the ARVC phenotype within the family has clearly been demonstrated. The female carriers of p.S358L presented a rather mild arrhythmogenic phenotype, whereas three male relatives died in the second to third decade by sudden cardiac death. In samples of additional 37 ARVC patients, 22 DCM patients, and 382 healthy controls, the mutation was not identified. Although the allele frequency appears to be low in German ARVC patients, *TMEM43* should be included in molecular genetic testing of ARVC patients.

Christensen AH, Andersen CB, Tybjærg-Hansen A, Haunso S, Svendsen JH (2011) Mutation analysis and evaluation of the cardiac localization of TMEM43 in arrhythmogenic right ventricular cardiomyopathy. Clin Genet 80:256–264

Klauke B, Kossmann S, Gaertner A, Brand K, Stork I, Brodehl A, Dieding M, Wahlhorn V, Anselmetti D, Gerdes D, Bohms B, Schulz U, zu Knyphausen E, Vorgerd M, Gummert J, Milting H (2010) De novo desmin mutation N116S is associated with arrhythmogenic right ventricular cardiomyopathy. Hum Mol Genet 19:4595–4607

Merner ND, Hodgkinson KA, Haywood AF, Connors S, French VM, Drenckhahn JD, Kupprion C, Ramadanova K, Thierfelder L, McKenna W (2008) Arrhythmogenic right ventricular cardiomyopathy type 5 is a fully penetrant, lethal arrhythmic disorder caused by a missense mutation in the TMEM43 gene. Am J Hum Genet 82: 809–821

Sen-Chowdhry S, Morgan RD, Chambers JC, McKenna WJ (2010) Arrhythmogenic cardiomyopathy: etiology, diagnosis, and treatment. Annu Rev Med 61: 233–253

**P 14 — Myocardial transcriptome analysis of human arrhythmogenic right ventricular cardiomyopathy (ARVC)**

Anna Gaertner^1^, Patrick Schwientek^2^, Peter Ellinghaus^3^, Holger Summer^3^, Stefan Golz^3^, Astrid Kassner^1^, Uwe Schulz^1^, Jan Gummert^1^, Hendrik Milting^1^

(1) Heart and Diabetes Center NRW, Erich & Hanna Klessmann-Institute for Cardiovascular Research & Development, Georgstr. 11, D-32545 Bad Oeynhausen, Germany

(2) Center for Biotechnology, Bielefeld University, Universitätsstr. 27, D-33615 Bielefeld, Germany

(3) Bayer Pharma AG, Global Drug Discovery, D-42096 Wuppertal, Germany

AGaertner@hdz-nrw.de; hmilting@hdz-nrw.de

Arrhythmogenic right ventricular cardiomyopathy (ARVC) is an inherited cardiomyopathy primarily of the right ventricle (Basso et al. 1996; Marcus et al. 1982; Thiene et al. 1988). Five of the causal genes are coding for desmosomal proteins (Gerull et al. 2004; McKoy et al. 2000; Norgett et al. 2000; Sen-Chowdhry et al. 2005; Syrris et al. 2007). Though electron microscopy of endomyocardial biopsies of ARVC patients revealed remodelling of intercalated discs and abnormal desmosomes, the molecular pathomechanisms are largely unknown (Basso et al. 2006).

Paired myocardial samples from the left (LV) and right ventricles (RV) were obtained from six non-failing (NF) donor hearts, six ARVC patients and seven patients with idiopathic dilated cardiomyopathy (DCM). The samples were analyzed by Affymetrix HG-U133 Plus 2.0 arrays for differential transcription. The analysis of our data by unsupervised cluster analyses and *t*-tests indicated that ARVC is a cardiomyopathy, which is not restricted to the RV. Unsupervised PCA of the ARVC subgroup revealed a common cluster for LV and RV samples of patients with desmosomal gene mutations. Surprisingly, in the LV of ARVC patients the gene expression of *Plakophilin-2* was shown to be up-regulated, whereas expression of *Desmocollin-2* was down-regulated. No transcriptional regulation of these genes was observed in the RV of ARVC patients in our study. None of the other ARVC-related desmosomal genes was regulated in RV or LV of ARVC patients.

Our study is the first analysis of specific ARVC-related RV and LV gene expression patterns in terminally failing human hearts. It offers further insight into the disease mechanisms of ARVC and might probably reveal further candidate genes relevant for molecular genetics of ARVC.

Basso C, Czarnowska E, Della Barbera M, Bauce B, Beffagna G, Wlodarska EK, Pilichou K, Ramondo A, Lorenzon A, Wozniek O, Corrado D, Daliento L, Danieli GA, Valente M, Nava A, Thiene G, Rampazzo A (2006) Ultrastructural evidence of intercalated disc remodelling in arrhythmogenic right ventricular cardiomyopathy: an electron microscopy investigation on endomyocardial biopsies. Eur Heart J 27:1847–1854

Basso C, Thiene G, Corrado D, Angelini A, Nava A, Valente M (1996) Arrhythmogenic right ventricular cardiomyopathy. Dysplasia, dystrophy, or myocarditis? Circulation 94:983–991

Gerull B, Heuser A, Wichter T, Paul M, Basson CT, McDermott DA, Lerman BB, Markowitz SM, Ellinor PT, MacRae CA, Peters S, Grossmann KS, Drenckhahn J, Michely B, Sasse-Klaassen S, Birchmeier W, Dietz R, Breithardt G, Schulze-Bahr E, Thierfelder L (2004) Mutations in the desmosomal protein plakophilin-2 are common in arrhythmogenic right ventricular cardiomyopathy. Nat Genet 36:1162–1164

Heuser A, Plovie ER, Ellinor PT, Grossmann KS, Shin JT, Wichter T, Basson CT, Lerman BB, Sasse-Klaassen S, Thierfelder L, MacRae CA, Gerull B (2006) Mutant desmocollin-2 causes arrhythmogenic right ventricular cardiomyopathy. Am J Hum Genet 79:1081–1088

Marcus FI, Fontaine GH, Guiraudon G, Frank R, Laurenceau JL, Malergue C, Grosgogeat Y (1982) Right ventricular dysplasia: a report of 24 adult cases. Circulation 65:384–398

McKoy G, Protonotarios N, Crosby A, Tsatsopoulou A, Anastasakis A, Coonar A, Norman M, Baboonian C, Jeffery S, McKenna WJ (2000) Identification of a deletion in plakoglobin in arrhythmogenic right ventricular cardiomyopathy with palmoplantar keratoderma and woolly hair (Naxos disease). Lancet 355:2119–2124

Norgett EE, Hatsell SJ, Carvajal-Huerta L, Cabezas JC, Common J, Purkis PE, Whittock N, Leigh IM, Stevens HP, Kelsell DP (2000) Recessive mutation in desmoplakin disrupts desmoplakin-intermediate filament interactions and causes dilated cardiomyopathy, woolly hair and keratoderma. Hum Mol Genet 9:2761–2766

Sen-Chowdhry S, Syrris P, McKenna WJ (2005) Genetics of right ventricular cardiomyopathy. J Cardiovasc Electrophysiol 16:927–935

Syrris P, Ward D, Asimaki A, Evans A, Sen-Chowdhry S, Hughes SE, McKenna WJ (2007) Desmoglein-2 mutations in arrhythmogenic right ventricular cardiomyopathy: a genotype–phenotype characterization of familial disease. Eur Heart J 28:581–588

Thiene G, Nava A, Corrado D, Rossi L, Pennelli N (1988) Right ventricular cardiomyopathy and sudden death in young people. N Engl J Med 318:129–133

**P 15 — Alpha-catenin recruits vinculin to cadherin junctions in a force-dependent manner: does it play a role during morphogenesis of the heart?**

Esteban Hoijman^1^, Iris Blonk^1^, Jeroen Bakkers^1^, Holger Rehmann^2^, Johan de Rooij^1^

(1) Hubrecht Institute, Utrecht, The Netherlands

(2) University Medical Center Utrecht, Utrecht, The Netherlands

e.hoijman@hubrecht.eu

Several animal models have shown that proteins of the cell–cell junctions (CCJs) such as VE-cadherin, alpha-catenin and vinculin are important for heart development. Next to their well-known structural role, these CCJs mediated by cadherins are also mechanosensitive. The exact role of the cadherin complex proteins during heart morphogenesis is not well-understood and, in particular, the role of mechanotransduction on this process is unknown.

Force-dependent recruitment of vinculin to CCJs is essential for cadherin mechanosensing. To determine the role of mechanotransduction in heart development, we aimed to block this recruitment. As it was proposed that vinculin recruitment depends on its binding to alpha-catenin, we replaced the vinculin interaction domain of alpha-catenin with the homologous domain from vinculin (its closest homolog), obtaining an alpha-catenin/vinculin hybrid. This construct fully restored the formation of CCJs in several alpha-catenin negative cell lines, and actomyosin contraction was no longer able to induce the recruitment of vinculin to the junctions in these cells. Our ultimate goal is to block the alpha-catenin/vinculin interaction in vivo during zebrafish heart development. To this end, we started analyzing the heart phenotypes produced by injection of vinculin and alpha-catenin morpholinos. Both of these showed different defects in heart development at 24 and 48 hpf. Next, we will test the defects caused by the specific loss of junctional vinculin, by rescuing alpha-catenin depletion with the alpha-catenin/vinculin hybrid.

In conclusion, the results in vitro suggest that force-dependent recruitment of vinculin to CCJs can be specifically prevented by an alpha-catenin mutant that does not perturb CCJs per se. This allows us to design in vivo experiments to determine the role of cadherin mechanosensing during heart morphogenesis.

